# Multi-level analysis of reproduction in an Antarctic midge identifies female and male accessory gland products that are altered by larval stress and impact progeny viability

**DOI:** 10.1038/s41598-020-76139-6

**Published:** 2020-11-13

**Authors:** Geoffrey Finch, Sonya Nandyal, Carlie Perretta, Benjamin Davies, Andrew J. Rosendale, Christopher J. Holmes, J. D. Gantz, Drew E. Spacht, Samuel T. Bailey, Xiaoting Chen, Kennan Oyen, Elise M. Didion, Souvik Chakraborty, Richard E. Lee, David L. Denlinger, Stephen F. Matter, Geoffrey M. Attardo, Matthew T. Weirauch, Joshua B. Benoit

**Affiliations:** 1 Department of Biological Sciences, University of Cincinnati, Cincinnati, OH USA; 2 Department of Biology, Mount St. Joseph University, Cincinnati, OH USA; 3 Department of Biology, Miami University, Oxford, OH USA; 4 Department of Biology and Health Science, Hendrix College, Conway, AR USA; 5 Departments of Entomology and Evolution, Ecology and Organismal Biology, The Ohio State University, Columbus, OH USA; 6 Center for Autoimmune Genomics and Etiology, Cincinnati Children’s Hospital Medical Center, Cincinnati, OH 45229 USA; 7 Department of Entomology and Nematology, University of California, Davis, Davis, CA 95616 USA; 8 Divisions of Biomedical Informatics and Developmental Biology, Cincinnati Children’s Hospital Medical Center, Cincinnati, OH 45229 USA; 9 Department of Pediatrics, University of Cincinnati College of Medicine, Cincinnati, OH 45267 USA

**Keywords:** Physiology, Reproductive biology, Ecology, Molecular ecology

## Abstract

The Antarctic midge, *Belgica antarctica*, is a wingless, non-biting midge endemic to Antarctica. Larval development requires at least 2 years, but adults live only 2 weeks. The nonfeeding adults mate in swarms and females die shortly after oviposition. Eggs are suspended in a gel of unknown composition that is expressed from the female accessory gland. This project characterizes molecular mechanisms underlying reproduction in this midge by examining differential gene expression in whole males, females, and larvae, as well as in male and female accessory glands. Functional studies were used to assess the role of the gel encasing the eggs, as well as the impact of stress on reproductive biology. RNA-seq analyses revealed sex- and development-specific gene sets along with those associated with the accessory glands. Proteomic analyses were used to define the composition of the egg-containing gel, which is generated during multiple developmental stages and derived from both the accessory gland and other female organs. Functional studies indicate the gel provides a larval food source as well as a buffer for thermal and dehydration stress. All of these function are critical to juvenile survival. Larval dehydration stress directly reduces production of storage proteins and key accessory gland components, a feature that impacts adult reproductive success. Modeling reveals that bouts of dehydration may have a significant impact on population growth. This work lays a foundation for further examination of reproduction in midges and provides new information related to general reproduction in dipterans. A key aspect of this work is that reproduction and stress dynamics, currently understudied in polar organisms, are likely to prove critical in determining how climate change will alter their survivability.

## Introduction

The Antarctic midge, *Belgica antarctica,* is long-lived, wingless, and the only insect endemic to maritime Antarctica^1,2^. It has a patchy distribution along the western coast of the Antarctic Peninsula and South Shetland Islands, where it may form large aggregations into the thousands under favorable conditions^1,2^. The larval period lasts 2 years; growth and development take place during the short austral summer, and larvae overwinter encased in a matrix of ice and substrate^3^. Larvae commonly reside in damp areas and feed non-selectively on dead plant and animal matter, algae, and microorganisms^4,5^. Larvae are extremely tolerant of numerous stresses including cold, dehydration, and UV exposure^6–10^. Adult emergence is a fairly synchronous event occurring in early summer^5^, and there is some evidence for protandry (males emerge before females)^5,11^. The wingless adults mate in swarms formed on rocks and other features of the substrate^5,11^. Environmental stressors in Antarctica can be severe and highly variable over short distances and time periods^12,13^, and swarming may play a role in locating and taking advantage of intermittent, favorable microhabitats^1,2,14^. Mating swarms present a potential obstacle for establishing long-lasting colonies in a laboratory setting, as mating is likely facilitated by large-scale, synchronous adult emergence^11^.

Adult females that emerge in the laboratory lay, with a few exceptions, a single egg mass, each containing 40–50 eggs^1,2,5,11^. Nevertheless, multiple mating events by females are common, and multiple ovipositions, albeit with reduced eggs, have been reported^2,5,11^. Specific underlying materials transferred from the male to the female during copulation are unknown in *B. antarctica*. Eggs are encased in a hygroscopic gel that has been suggested as a potential food source for developing larvae^5,11^. This gel is likely secreted by the female accessory gland during oviposition, but the protein components of the gel are unknown. Little additional information is available on reproduction in these extremophiles^5,11^, and chironomid reproduction in general is poorly studied beyond the basic descriptions of their reproductive anatomy and impaired reproduction during exposure to toxic substances^15–17^.

By contrast, reproduction in other dipteran families has been examined extensively^18–30^. In most Diptera, insemination does not involve injection of a spermatophore; rather, male seminal fluid is usually transferred directly into the female reproductive tract, often with the addition of a mating plug to reduce male-male competition as is seen in mosquitoes and *Drosophila*^31–34^. Some flies do utilize a spermatophore, which may facilitate quicker mating while also creating a barrier to multiple inseminations^35^. In the tsetse fly, *Glossina morsitans,* a dipteran that uses a spermatophore, proteins making up the spermatophore are secreted from the male accessory gland during copulation^34,36^. The spermatophore is deposited such that the spermatozoa are funneled efficiently to the openings of the spermathecal ducts, allowing only one spermatophore to maintain this connection at a time^34,36^. In mosquitoes, accessory gland-specific proteins, along with the steroid hormone, 20-hydroxy-ecdysone, are transferred to the female during copulation, producing a mating plug^27,33,37^. In *An. gambiae* the mating plug has diverse effects that promote copulation, enhance egg production, and even trigger egg laying^27,33,38,39^. First-male precedence and last-male precedence have both been reported in multiple species within the Diptera^40–43^, but it remains unknown how fertilization priority is established in *B. antarctica*. Depending on the mating strategy of the species, contents of the spermatophore may include a large amount of seminal fluid proteins (SFPs) and other factors or contain primarily the sperm^21,31,34,37^. Seminal fluid proteins are suspected or have been demonstrated to have a variety of functions that serve the goal of increasing male fertility^,^^21,29,31,40^, thus impairment can compromise sperm function and the ability to fertilize. The particular cocktail and amounts of SFPs utilized reflects the selection pressure from life-history differences, conspecific competition, and diverse reproductive strategies between species and even within species^25,26,44,45^.

Secretions from the female accessory glands also play important roles in insemination and oviposition in dipterans, as well as other insects. In the house fly, *Musca domestica,* female accessory gland secretions enhance sperm viability^46,47^. In some species, substances secreted from the female accessory gland are expelled with the eggs at oviposition and function as an adhesive, anchoring eggs to the substrate^48^. Accessory gland secretions may also provide protection from diverse biotic and abiotic stressors. In the medfly, *Ceratitis capitata*, the primary components of the accessory gland secretion deposited during oviposition are ceratotoxins, which act as potent antibacterial agents^49^. Similarly, accessory gland secretions from the sand fly, *Phlebotomus papatasi*, have antimicrobial effects that protect the eggs, developing embryos, and adult female reproductive tract from microbial invasion^50^. Some male seminal fluids contain antimicrobial peptides, probably for similar reasons as in the female^51,52^. Female accessory gland proteins can also be a source of nutrition for developing progeny, either while growth occurs in the mother or as a secreted food source to nourish free living offspring^22,53–55^.

In this study, we used RNA-seq, proteomics, and functional analyses to examine the reproductive physiology of *B. antarctica*. Specifically, male and female accessory glands were examined to identify factors related to male accessory protein generation and synthesis of egg components during oviposition. Proteomic analysis of the gel secretion was used to identify its components, while comparative genomic analyses was used to identify orthologs of specific reproduction-associated genes in mosquitoes and midges. Functional studies revealed that stress impinging on late instar larvae impacts synthesis of gene products associated with reproduction, lowering both male and female reproductive success. Furthermore, we determined that the gel likely acts to prevent egg dehydration and serves as a thermal buffer, preventing overheating of the eggs. Population growth modeling revealed that impaired fecundity from larval stress may reduce reproduction below population replacement levels. Our analysis showed that reproduction in the Antarctic midge is directly impacted by larval stress, and identified novel roles for products manufactured by the female accessory gland. These studies confirm that an understanding of reproductive biology is critical for establishing how these Antarctic extremophiles are able to survive and proliferate in the challenging polar environment.

## Results

### Description of mating and reproductive organs in *B. antarctica*

**Figure 1:**
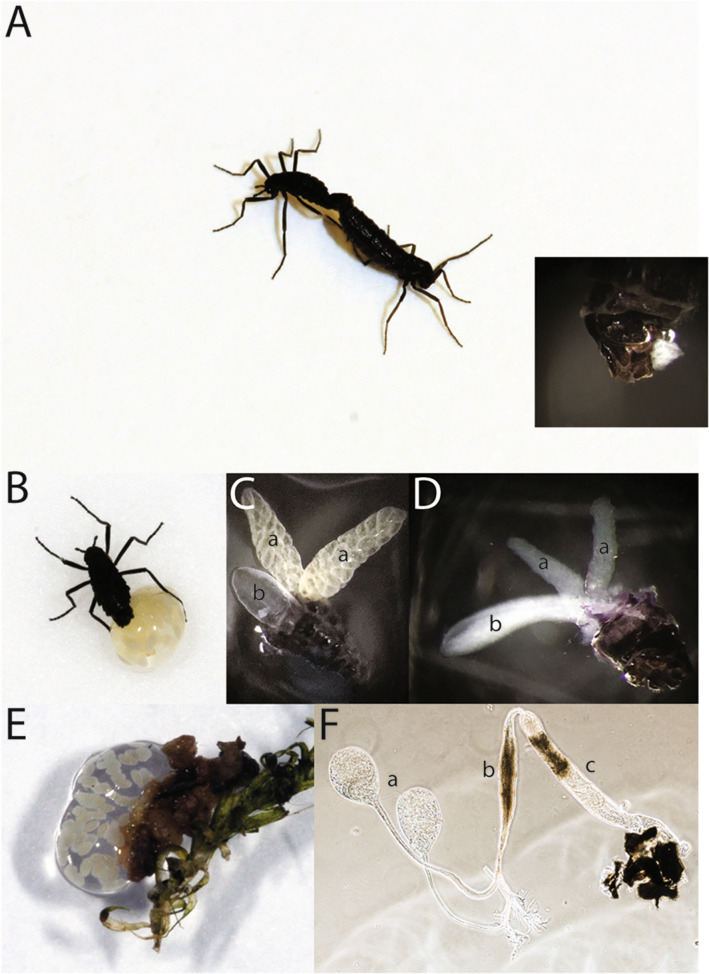
Antarctic midge, *Belgica antarctica*, during reproduction. (**A**) Mating pair, male on left. Inset, spermatophore transferred to females immediately after copulation. Image is posterior end of female and white material is the spermatophore. (**B**) Female depositing eggs and accessory gland-derived gel. (**C**) Accessory gland (left middle, b) and ovaries (top left and right, a) of gravid females 3 days after adult eclosion. (**D**) Female accessory glands (left, a) and ovaries (top and right, b) following egg and gel deposition. (**E**) Egg mass following the completion of deposition. (**F**) Male reproductive tract, a. testes, b, accessory gland, and c, common duct.

Beyond copulation and sex ratios^2,5,11^, little is known about mating and reproductive aspects of *B. antarctica.* In this study, the reproductive organs were observed by dissection. Following copulation (Fig. 1A), a spermatophore, rather than a mating plug, containing sperm and other products, most like derived from the accessory gland, are transferred as a bundle to the females (Fig. 1A, inset). Females deposit a gel around the fertilized eggs, and it is within this gel that embryogenesis occurs (Fig. 1B). The source of the gel appears to be the female’s accessory gland (Fig. 1C) because the gland is depleted followed egg laying (Fig. 1D,E). The male reproductive organs, including the testes and accessory glands, are depicted in Fig. 1F. The general organization and structure of the male and female reproductive anatomy is similar to that of another midge, *Chironomus plumosus*^15^ and mosquitoes^56^, with the exception that the female accessory gland of *B. antarctica* is dramatically enlarged.

### RNA-seq analyses of *Belgica* reproduction

**Figure 2:**
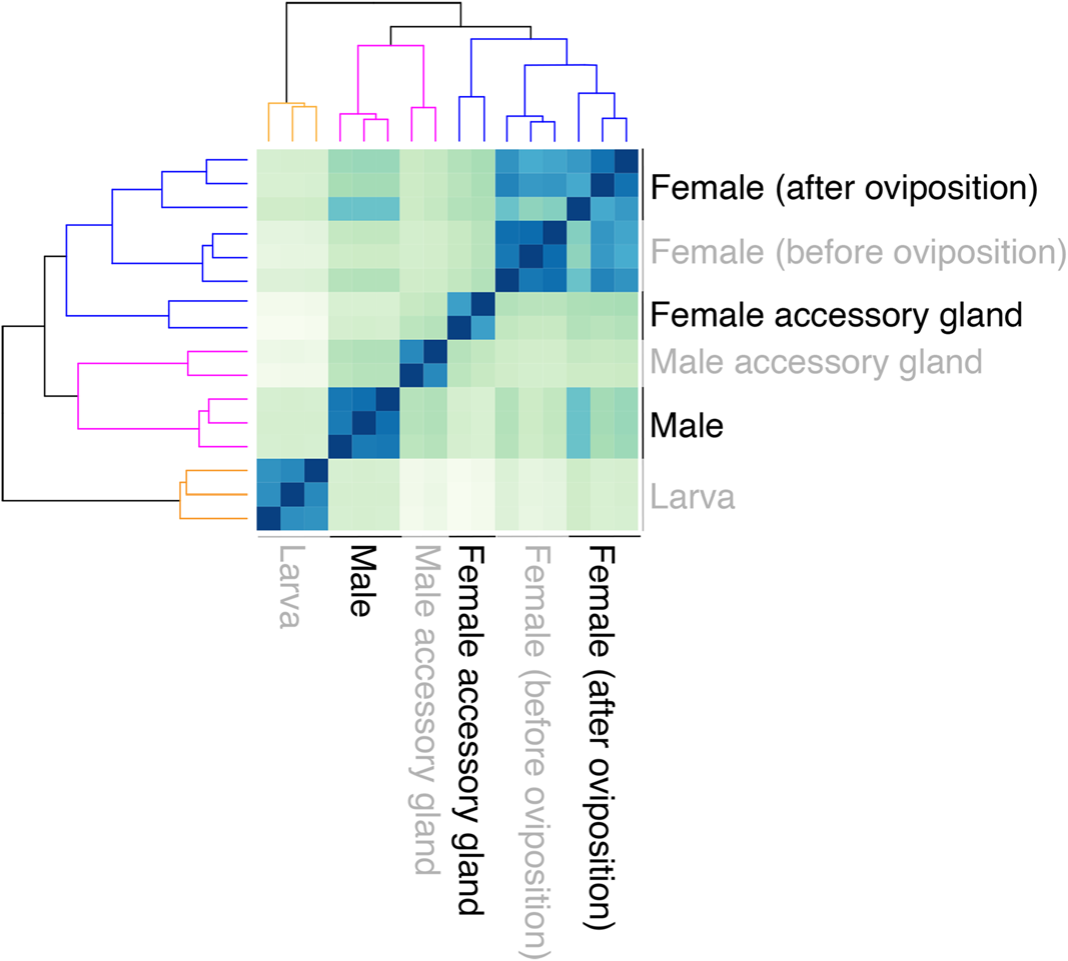
Gene expression heat map of Antarctic midge, *Belgica antarctica*, during development, between sexes, and for accessory glands. Hierarchical clustering of RNA-seq gene expression patterns for males, females, larvae, and accessory glands based on sample distance (Euclidean distance matrix) of differentially expressed contigs.

Differential transcript levels were determined for males, females, and larvae using two independent pipelines and the *B. antarctica* genome as a reference^57^. The two pipelines yielded 95% overlap at our significance cut-off (two-fold difference, combined transcripts per million (TPM) among all samples of at least 5, and a correction-based P-value < 0.05). Between 60 and 71% of the reads from each RNA-seq set mapped to predicted genes. At the four-fold or greater level for stage-specificity, overlap between the two methods exceeded 99%; thus we used the CLC Genomics (Qiagen) pipeline based on previous studies^36,58^ for the remaining analyses (Table S1). To determine the relative similarities and differences in gene expression between the life stages and tissues, the samples were clustered by differential gene expression patterns using a Euclidean distance matrix. The transcriptomes cluster primarily based on sex. The male and female accessory gland libraries bear greater similarity to respective sex specific libraries from whole insects. The larval libraries were most divergent from the whole and tissue specific adult libraries (Fig. 2). When the de novo assembly was examined for presence of putative microbial symbionts, there were 66 putative contigs that could not be directly linked to *B*. *antarctica* through BLAST comparison to the genome (Table S2). Of these novel contigs, expression levels were extremely low in at least one specific female accessory gland RNA-seq set (therefore not consistent in expression as would be expected for an obligate symbiont) or there was a match in another insect system (Table S2). This suggests a distinct absence of bacteria in the female accessory gland (Table S2), indicating the gel does not likely serve as a source of potential microbial symbionts as in other insects^53^.

**Figure 3:**
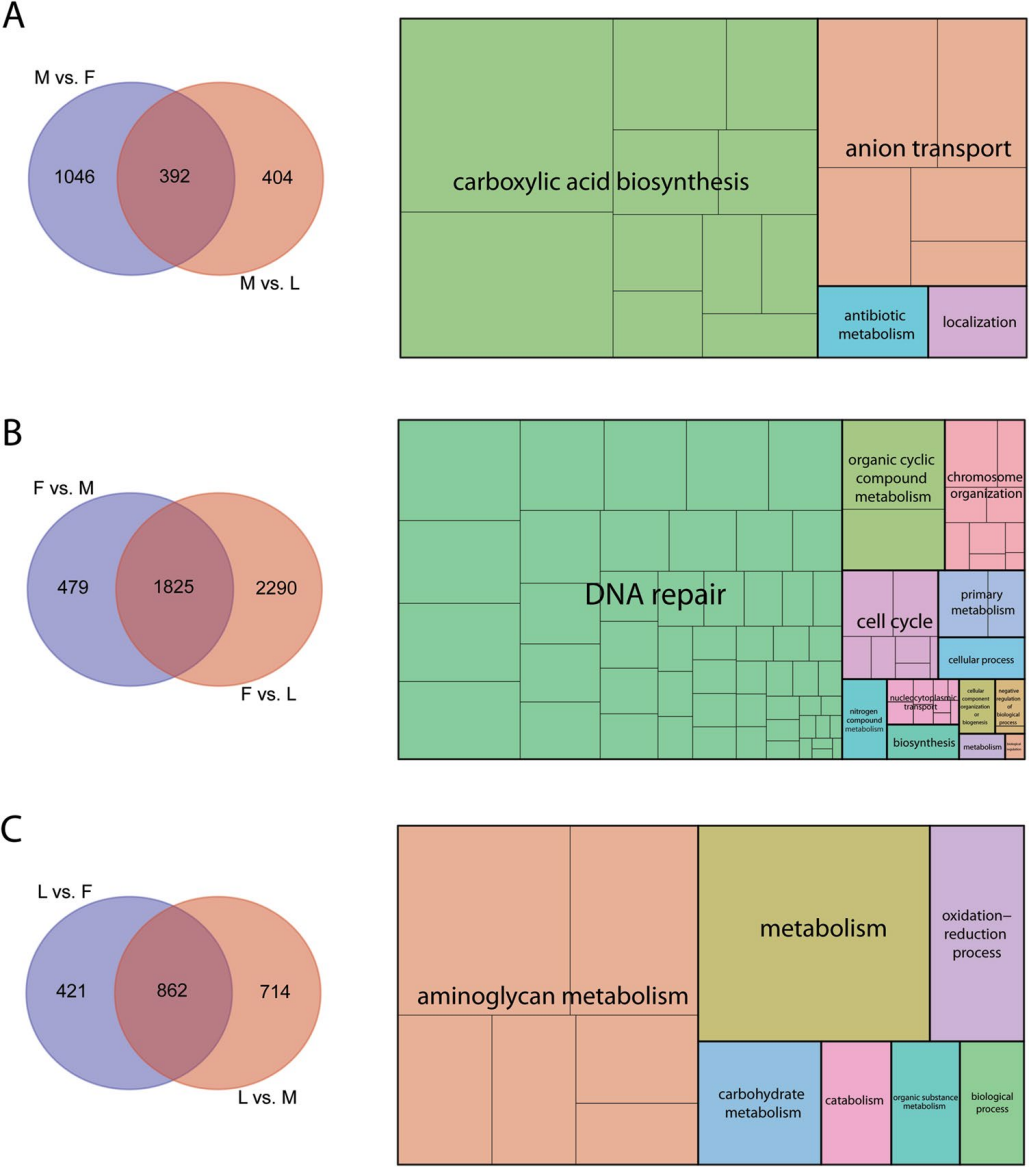
Genes uniquely enriched for the Antarctic midge, *Belgica antarctica* in males, females, and larvae and associated gene ontology enrichment. (**A**) Gene enriched in males (left) and gene ontology (right), (**B**) gene enriched in females (left) and gene ontology (right), (**C**) gene enriched in larvae (left) and gene ontology (right). Each box represents a specific category and color represent major GO groups. F, female; M, male; L, larvae.

**Figure 4:**
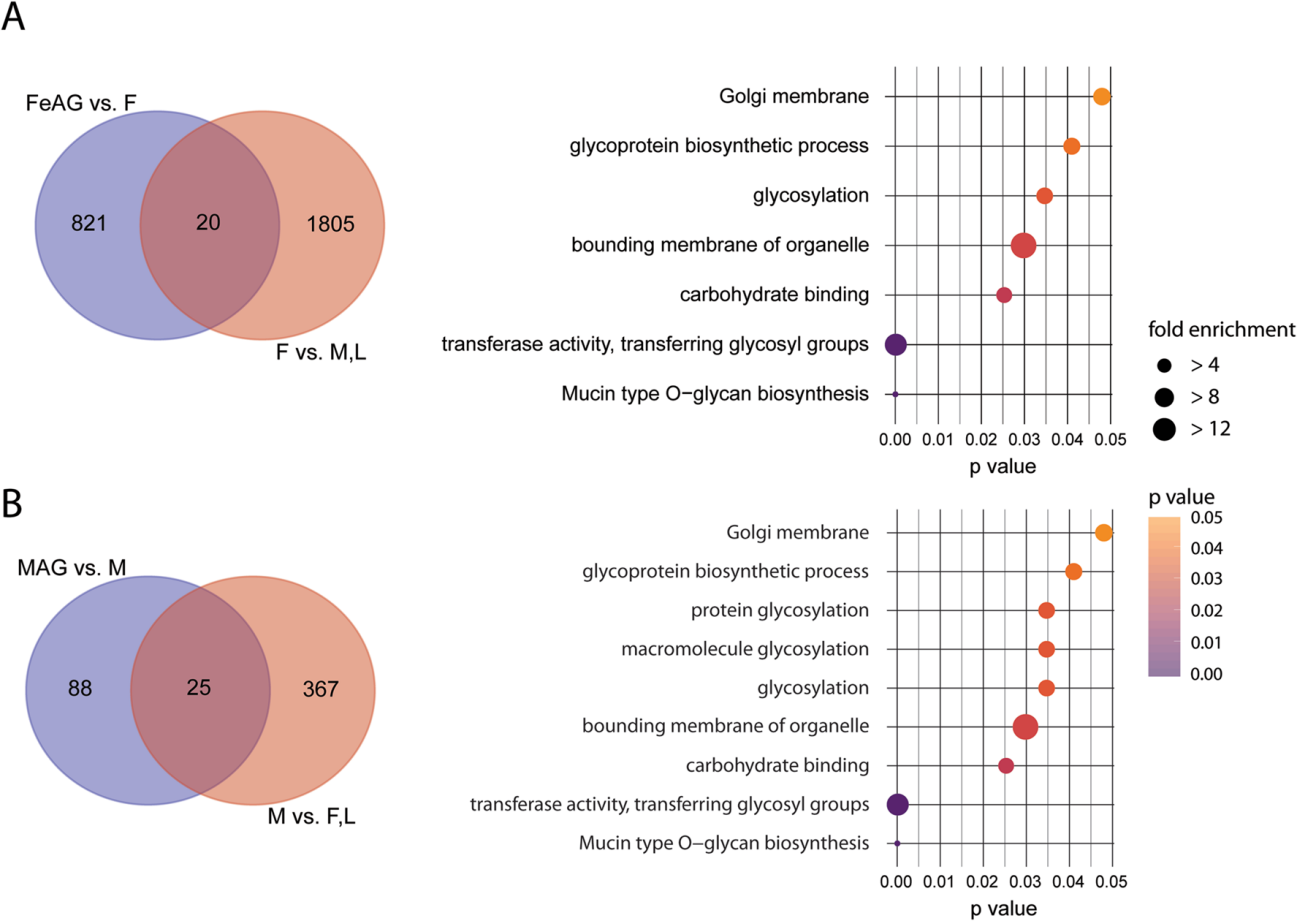
Genes uniquely enriched in the Antarctic midge, *Belgica antarctica*, female and male accessory glands and associated gene ontology enrichment. (**A**) Genes enriched in female accessory glands (left) and gene ontology (right), (**B**) genes enriched in male accessory gland (left) and gene ontology (right). GO conducted with g:Prolifer^59^. F, female; M, male; L, larvae; MAG, Male accessory glands; FeAG, female accesspry glands.

Individual comparisons between stages revealed 392 male-enriched genes, 1825 female-enriched genes, and 862 genes enriched in larvae, where enrichment was based on the specific genes having significantly higher expression in each sample when compared to all other samples (Fig. 2, Tables S3–S5). Specific gene ontology (GO) categories enriched in each stage included carboxylic acid biosynthesis in males, DNA repair in females, and aminoglycan metabolism in larvae (Fig. 3A–C, Tables S3–S5). When accessory glands were specifically examined, 20 genes enriched in the female accessory gland had significantly higher expression in females compared to other stages (Fig. 4A, Table S6). A similar analysis for the male accessory gland identified 25 enriched genes. GO categories associated with the female accessory gland were associated with glycosylation and mucin biosynthesis. Notably, similar GO categories were documented in the gene products of the male accessory gland (Fig. 4B, Table S7).

**Figure 5:**
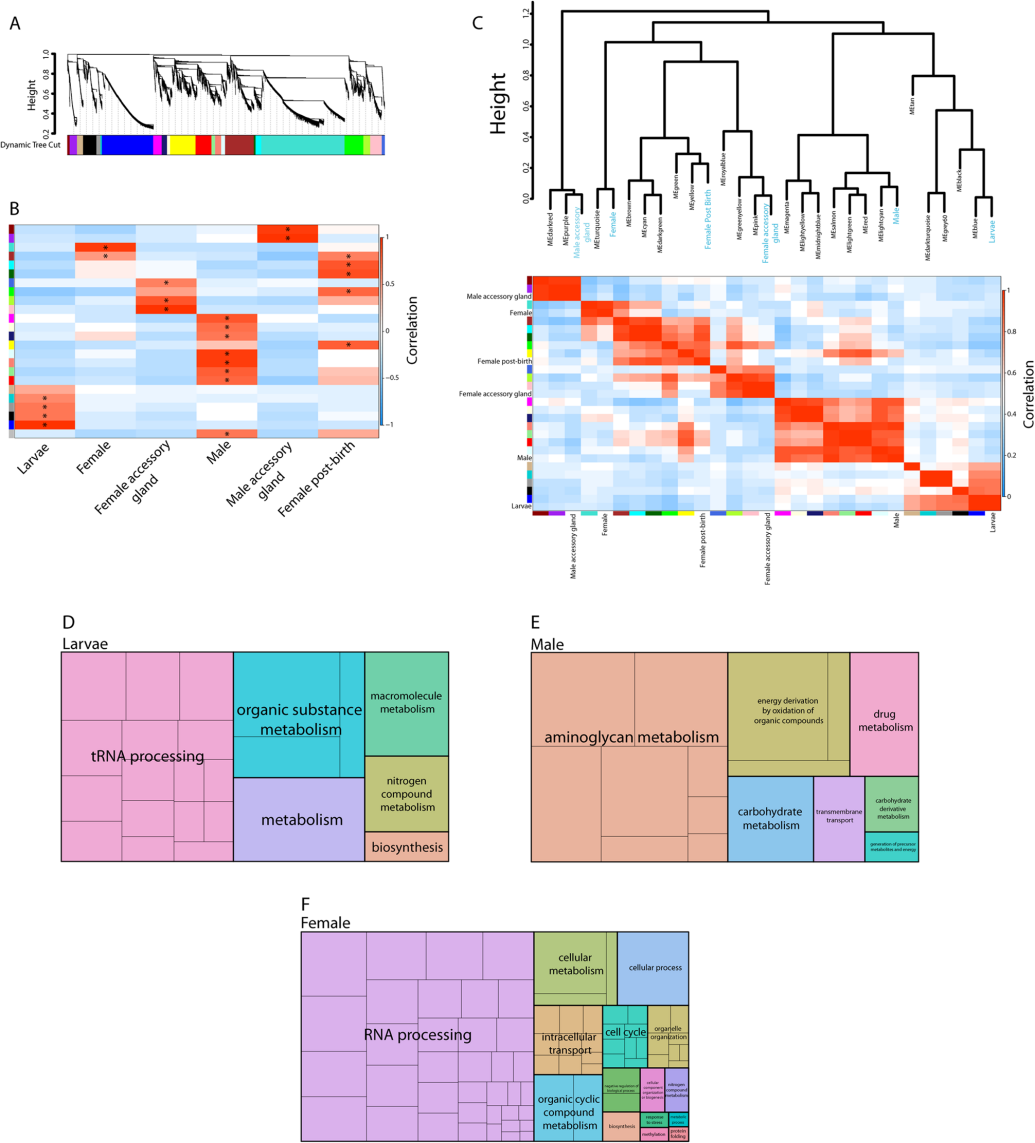
Weighted gene co-expression network analysis (WGCNA) for larvae, adults, or for male and female accessory glands of the Antarctic midge, *Belgica antarctica*. (**A**) Average linkage hierarchical clustering dendrogram of the genes. Modules, designated by color code, are branches of the clustering tree. (**B**) Correlation of module eigengenes to larvae, adults, and accessory gland traits. Each row corresponds to a module eigengene and columns are traits. *Represents values with a significant positive correlation for Pearson r (P < 0.05). (**C**) Unsupervised hierarchical clustering heatmap (bottom) and dendrogram (top) of module eigengenes and traits. Gene ontology (GO) analysis of eigengenes associated with larvae (**D**), males (**E**), and females (**F**). GO conducted with g:Prolifer^59^ and visualized with REVIGO^61^. Colors for modules between (**A**–**C**) are the same. Color patterns in (**D**–**F**) is random and size of the boxes represents the relative number of each specific GO categories.

Beyond this first analysis, weighted correlation network analysis (WGCNA^60^) was used to identify clusters (modules) of highly correlated genes between larval and adult stages, along within specific reproductive organs (Fig. 5, Tables S8–S12). Specific modules of enriched expression were identified in each tissue, larvae, and adults (Fig. 5B,C). Unique GO categories were noted for larvae, females, and males (Fig. 5). Female accessory glands had few enriched GO categories based on WGCNA results; enriched categories included phosphatase binding, response to stimulus, and ion channel activity. Male accessory glands also had a low number of GO categories associated with enriched modules; these included metallopeptidase activity and integral components of the membrane. For larvae, males, and females, GO categories from stage specific modules showed overlap with our previous analysis (Fig. 3), but unique categories were also identified (Fig. 5).

### Proteomic and transcriptional analyses of accessory gland derived gel

**Figure 6:**
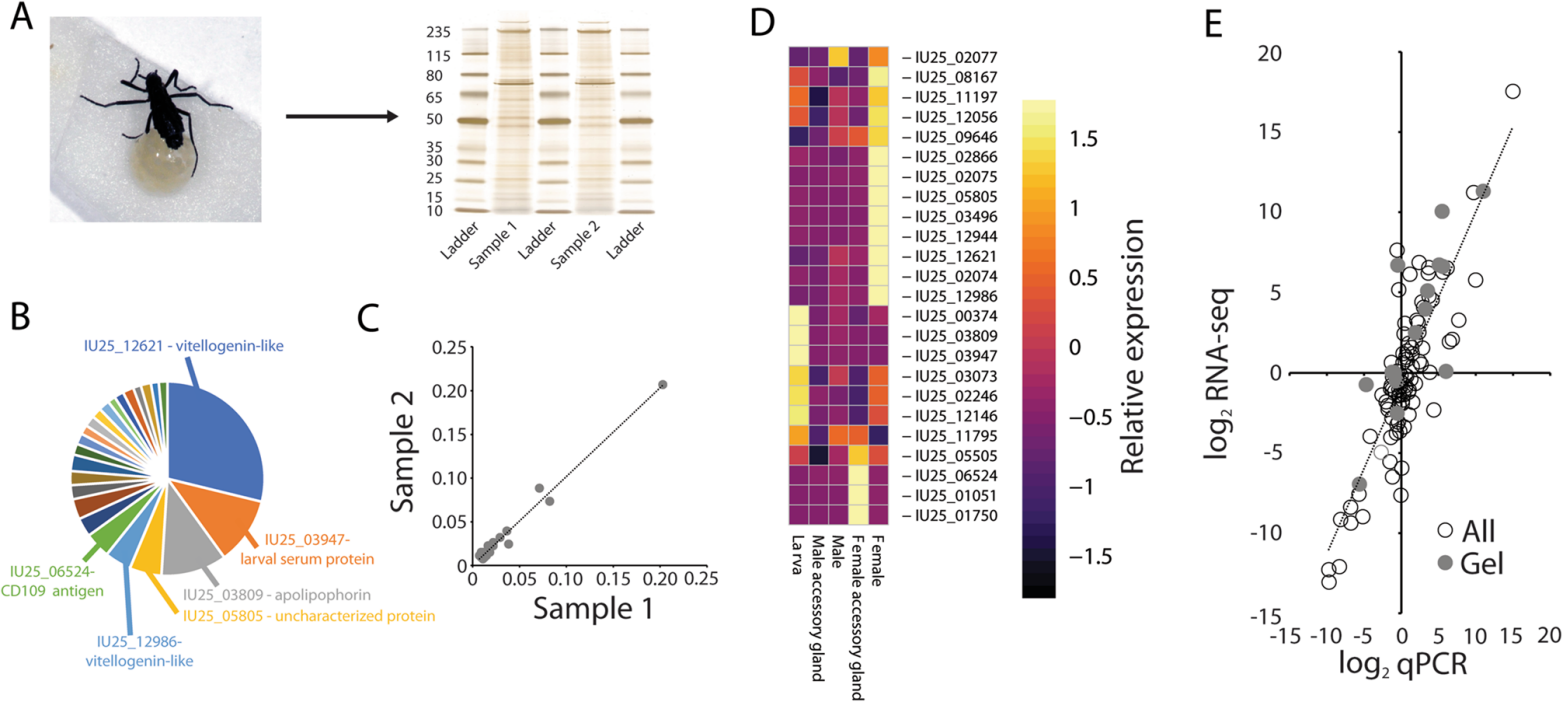
Proteomic analysis of female accessory gland derived gel material from the Antarctic midge, *Belgica antarctica*. (**A**) Female depositing eggs with gel and protein components of two gel samples without eggs. Original gel images in Fig. S1. (**B**) Identification of proteins that represent at least 3% of total protein composition of the accessory gland gel. (**C**) Congruence of protein abundance and content between two gel samples. (**D**) Heatmap for transcript levels of gel-specific genes among larvae, females, males, and accessory glands. (**E**) qPCR validation of RNA-seq data. All tested genes have Pearson correlation coefficients over 0.85. Gel specific genes have a Pearson correlation of 0.87.

Proteomic analysis of the gel surrounding the eggs revealed 24 associated proteins (Fig. 6). Three proteins comprised a majority of the gel (Fig. 6B, Table S13): a vitellogenin-like protein (IU25_12621), larval serum protein (IU25_03947), and an apolipophorin (IU25_03809). The two analyzed gel samples had a highly-correlated protein content (Pearson correlation = 0.92, Fig. 6C). RNA-seq analyses revealed that transcripts for most of the gel proteins are not directly generated in the female accessory gland (Fig. 6D), but instead are produced in other female organs (e.g. IU25_12621) or within the larvae (e.g. IU25_03947 and IU25_03809). qPCR validation of the protein gel components confirmed that transcripts for each of the gel proteins are expressed in tissues identified in the RNA-seq studies (Fig. 6E). Additional qPCR analyses for genes not involved in producing major components of the gel served as validation for results obtained from other RNA-seq samples (Fig. 6E).

### Transcriptional regulation of reproductive-associated factors

**Figure 7:**
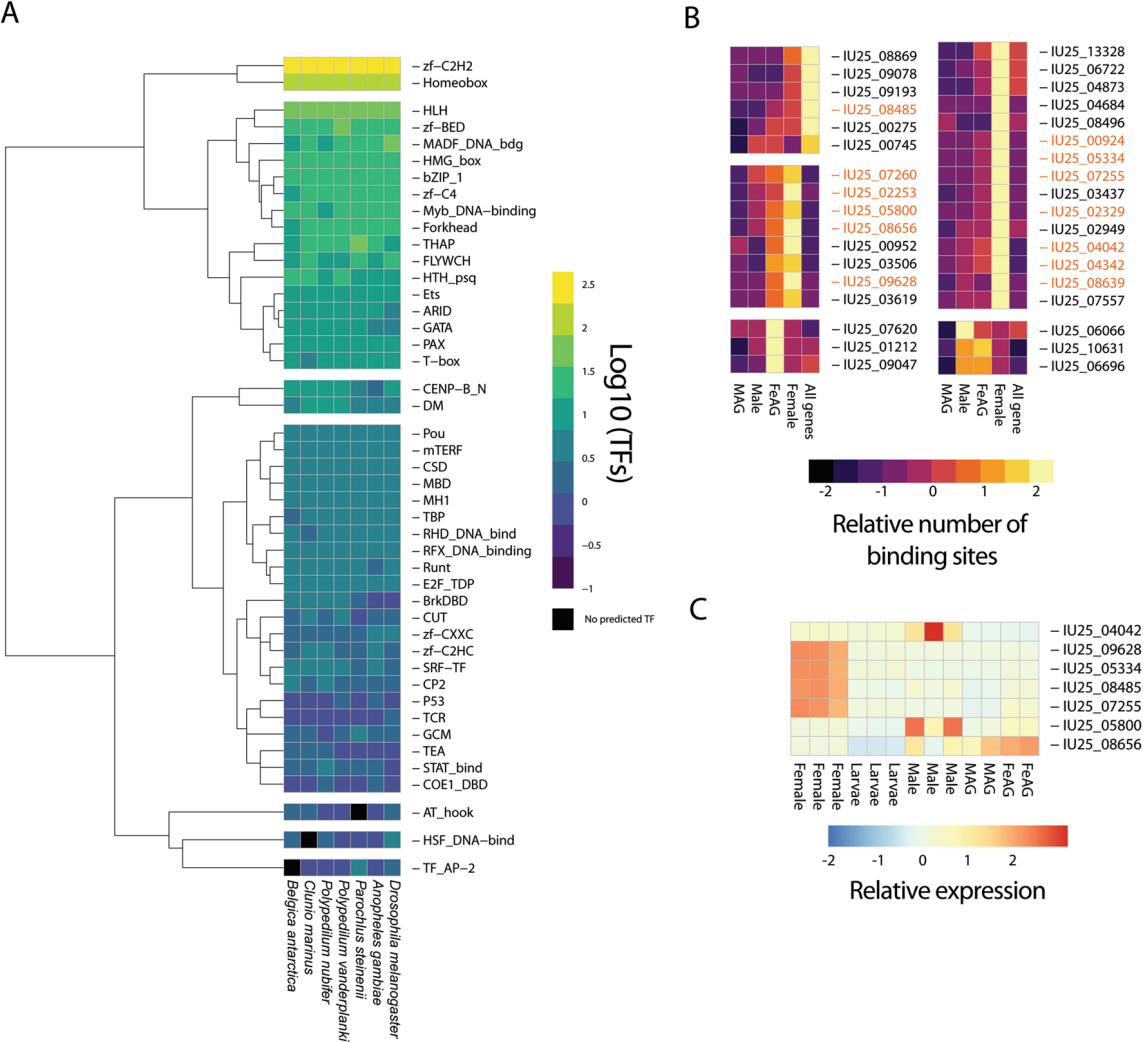
Transcription factors (TFs) and TF binding sites associated with reproduction in the Antarctic midge, *Belgica antarctica*. (**A**) Relative abundance of transcription factors encoded by genomes of midges and mosquitoes. Assignment of specific TFs to families is present in Table S14. TF families towards the top contain more TFs in *B. antarctica*. (**B**) Enrichment for binding site motifs for specific TFs in regulatory regions (2000 bp; 500 bp data not shown due to overlap with 2000 bp) of genes expressed highly in specific stages and accessory glands. Groups of TFs are separated by their motif enrichment profiles across samples. Those highlighted in orange are significantly enriched within the specific stage or tissue. Scale for heatmap is set at relative abundance on a Z scale of − 2 to 2 across each row. (**C**) Transcript levels of select TFs with significant motif enrichment in the promoters of genes expressed in specific tissues (orange font color in (**B**)). Scale for heatmap is set at relative abundance on a Z scale of − 2 to 2 across each row. *MAG* male accessory gland, *FeAG* female accessory glands.

To compare potential mechanisms regulating gene expression between samples, we predicted putative transcription factors (TFs, Fig. 7A, Table S14) and their DNA binding motifs. We next performed TF binding site motif enrichment analysis in the putative upstream regulatory regions of our differentially expressed gene sets (see “Materials and methods” section). This analysis revealed that fourteen TFs had significantly enriched binding sites in at least one gene set (Fig. 7B). Of these, one TF showed enhanced binding in the 500 bp regulatory regions of male-enriched genes. The remaining thirteen showed enhanced binding in female-enriched genes; we limited our analysis to the set of seven TFs with enhanced binding in both 500 and 2000 bp regions of the female- and/or FeAG-enriched gene sets. Five members of this set showed higher transcript levels in either the female or female accessory gland compared to other tissues, larvae, or adults (Fig. 7C). The increased gene expression in the respective tissues or stage along with enriched binding sites suggest that these TFs are key regulatory elements for female reproduction in *B. antarctica*. Transcription factors include forkhead box protein 1 and mothers against decapentaplegic (Mad) homolog 1, which is critical to embryonic development^6^. Several transcription factors identified in our screening remain uncharacterized with no assigned biological functions.

### Comparative analyses between anopheline mosquitoes and chironomid midges

**Figure 8:**
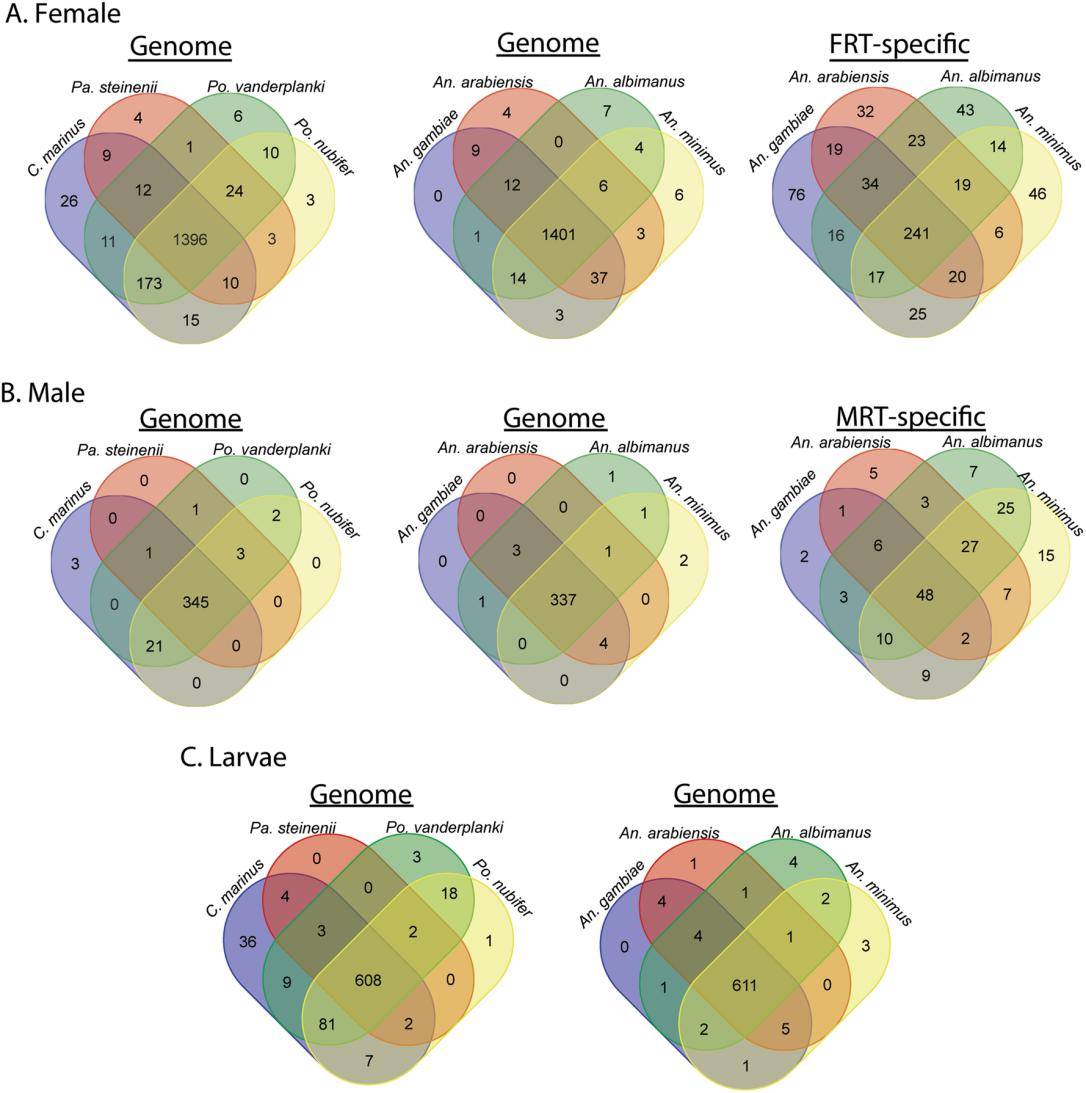
Comparative analysis of female, male, and larvae-specific gene sets with mosquitoes and midges to Antarctic midge, *Belgica antarctica*. (**A**) Female-specific genes compared to midges (left), mosquitoes (middle), and genes with enriched expression in the female reproductive tract (FRT) of mosquitoes (right)^25^. (**B**) Male-specific genes compared to midges (left), mosquitoes (middle), and genes with enriched expression in the male reproductive tract (MRT) of mosquitoes (right)^25^. (**C**) Larvae-specific genes compared to midges (left) and mosquitoes (right). Protein sequences were defined as orthologs if they had reciprocal-best BLASTp hits with an e-value < 10^−10^.

**Figure 9:**
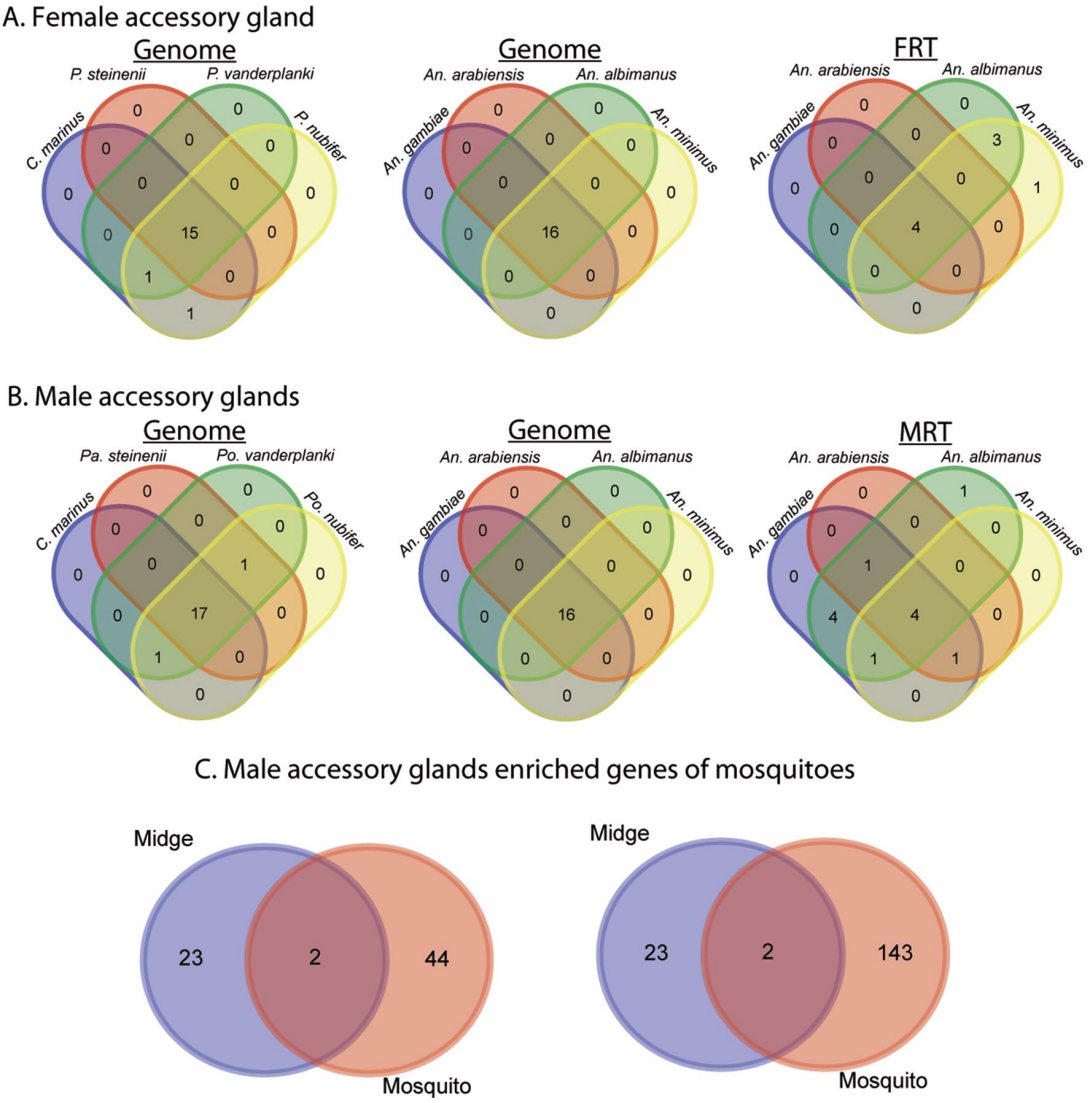
Comparative analysis of accessory gland gene sets with mosquitoes and midges to the Antarctic midge. (**A**) Female accessory gland genes compared to midges (left), mosquitoes (middle), and genes with enriched expression in the female reproductive tract of mosquitoes (right)^25^. (**B**) Male accessory gland genes compared to midges (left), mosquitoes (middle), and genes with enriched expression in the male reproductive tract of mosquitoes (right)^25^. (**C**) Overlap between genes expressed in male accessory glands between mosquitoes and *B. antarctica*. Left, highly enriched in *Anopheles* male accessory gland. Right, enriched in *Anopheles* male accessory gland. Enrichment for *Anopheles* male accessory gland genes is based on values from Izquierdo et al.^26^. Protein sequences were defined as orthologs if they had reciprocal-best BLASTp hits with an e-value < 10^−10^.

Sex- and tissue-specific gene sets from *B. antarctica* were compared with predicted gene sets from four species of *Anopheles* mosquitoes and four species of chironomid midges (Figs. 8, 9). Additionally, *B. antarctica* enriched gene sets were compared with gene sets enriched in male and female reproductive tracts of the same four *Anopheles* mosquitoes^25^ (Figs. 8, 9) as well as the male accessory gland enriched genes from five *Anopheles* species^26^.

Genes enriched in females of *B. antarctica* showed the highest degree of conservation among all species examined by a large margin. All five species of midges had 1396 genes in common, 1401 were shared with all four *Anopheles* species, and 1267 were common to all 9 species examined (Fig. 8). Gene ontology analysis of this set revealed enrichment in functions related to RNA, DNA, chromatin binding, nuclease activity, chromosome and organelle organization, transport of RNA and proteins in and out of the nucleus, RNA processing, and cell cycle regulation. No female-enriched genes detected in *B. antarctica* were unique when compared to the other eight species examined. *Belgica* females have 122 genes with no orthologues in any of the other four midge species. However, this gene set did not show significant enrichment in any GO functional categories.

Among males, 345 genes were common among midge species, and 337 genes were shared between *B. antarctica* and all four mosquito species (Fig. 8). Genes common to all midge species were enriched in functions associated with anion transport, alpha-amino acid catabolism, and carboxylic acid transmembrane transport. As with females, no genes were identified in the male-enriched gene set that were unique to *B. antarctica*. There were 16 genes unique to *B. antarctica* among the five midge species, but, again, they were not significantly enriched in any single functional category.

The larva-enriched gene set was compared to the midge and mosquito gene sets. Over 600 genes were identified with orthologues common to all examined midge and mosquito species (Fig. 8). The gene set common to all midge species was enriched in functional categories of chitin metabolism, iron ion binding, and cytochrome P450-driven metabolism. Two identified genes were unique to larvae of *B. antarctica* (Fig. 8, Table S5): one is uncharacterized, and the other putatively encodes polyubiquitin B. Gene ontology analysis comparing larvae of *Belgica* with other midges revealed 88 unique genes, but no functional categories were significantly enriched for this gene set.

Gene sets specifically enriched in male and female reproductive tracts of each mosquito species were compared to sex-specific gene sets in *B. antarctica* (Fig. 9; Tables S6, S7). Comparison of the female-enriched gene set with the female reproductive tract (FRT)-specific gene set yielded 241 genes with putative FRT orthologues. This set was particularly enriched in GO categories related to mitotic cell cycle processes and regulation of gene expression. For example, several orthologues in this set code for cyclins, cyclin dependent kinases, zinc-finger proteins, transcription factors, and transcription termination factors. Additionally, a large number of genes in this set (~ 44%) mapped to apparently uncharacterized orthologs. The comparison between male-enriched and Male Reproductive Tract (MRT) specific genes yielded a common set of 48 genes. This set is dominated by CLIP-domain serine proteases and cytochrome p450s, and also contains three glucose-dehydrogenases (Fig. 9). When *B. antarctica* MAG-enriched genes are compared to those for mosquitoes, only four overlapping genes were identified based on moderate or high expression reported in a previous study^26^ of the mosquito MAG (Fig. 9C); these were identified as venom allergen, carboxypeptidase, serine protease, and a gene of unknown function.

### Dehydration reduces larval serum protein and possibly subsequent egg production

**Figure 10:**
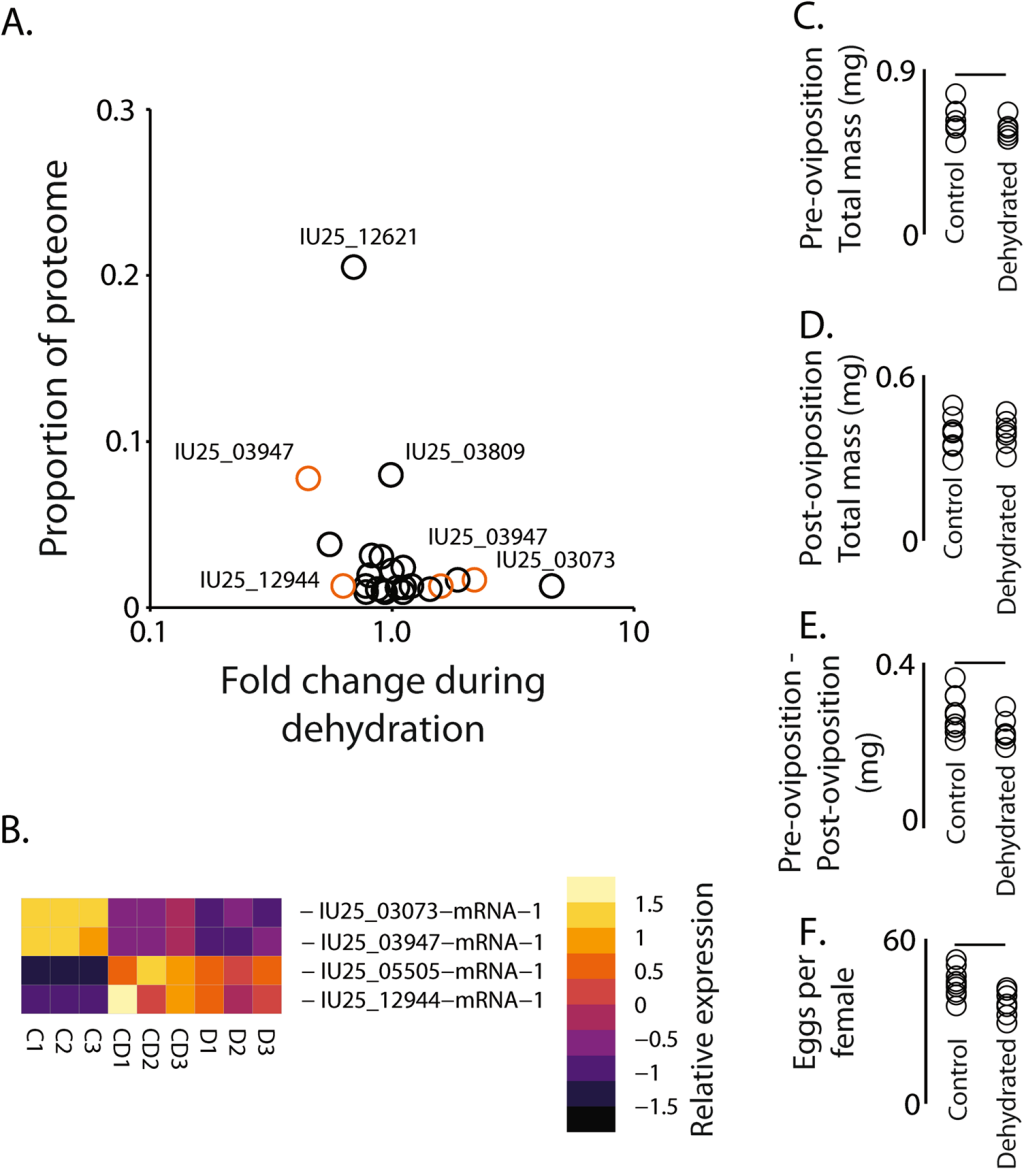
Expression changes in female accessory gland gel-associated proteins in larvae following dehydration stress. (**A**) Transcript level changes in larvae for gel proteins following dehydration stress. RNA-seq for dehydrated larvae were acquired from Teets et al.^62^. Orange denotes significance between control and dehydrated larvae based on RNA-seq analyses (FDR < 0.05). (**B**) Heat map of transcript levels for gel-associated proteins during dehydration (D) and cryoprotective dehydration (CD) compared to control (C) that are components of the gel proteome and significantly altered by dehydration. (**C**) Total mass before birth, (**D**) total mass after, (**E**) total mass difference between before and after borth, and (**F**) total egg production in females when control (non-dehydrated) and dehydrated larvae were allowed to complete development. T-test was utilized to examine statistical differences with the use of R statistic packages. Bars above indicate significance at P < 0.05.

Since specific female accessory gland components are expressed in larvae, we asked whether larval dehydration impacts expression of adult female accessory gland components (Fig. 10). To do so, we re-analyzed results from a previous RNA-seq study^62^ on dehydration and cryoprotective dehydration of *B. antarctica* (Fig. 10). In general, our analyses revealed similar results in relation to dehydration stress as the previous analysis^62^, with increased expression of genes in autophagic pathways and decreased levels of those linked to carbohydrate energy metabolism. One of the major female accessory gland proteins (larval serum protein, IU25_03947) had significantly reduced expression when larvae experienced either dehydration or cryoprotective dehydration (water loss specifically induced by cold temperatures) (Fig. 10A,B). This protein is a component of the accessory gel and, as a hexamerin, serves as a protein reserve for subsequent developmental stages^63–65^. The dehydration-evoked suppression in transcripts that underlie reproductive-associated factors possibly results in reduced amounts of materials invested in the progeny at birth (Fig. 10C–E, t-test: t-statistic_1,15_ = 3.46: P < 0.05) and reduced egg output (Fig. 10F, t-test: t-statistic_1,14_ = 3.01: P < 0.05). These results suggest that stress experienced as larvae may have a direct impact on female reproduction, most likely acting through reduced expression of larval serum protein.

### Dehydration lowers specific male accessory gland components and impacts fertilization

**Figure 11:**
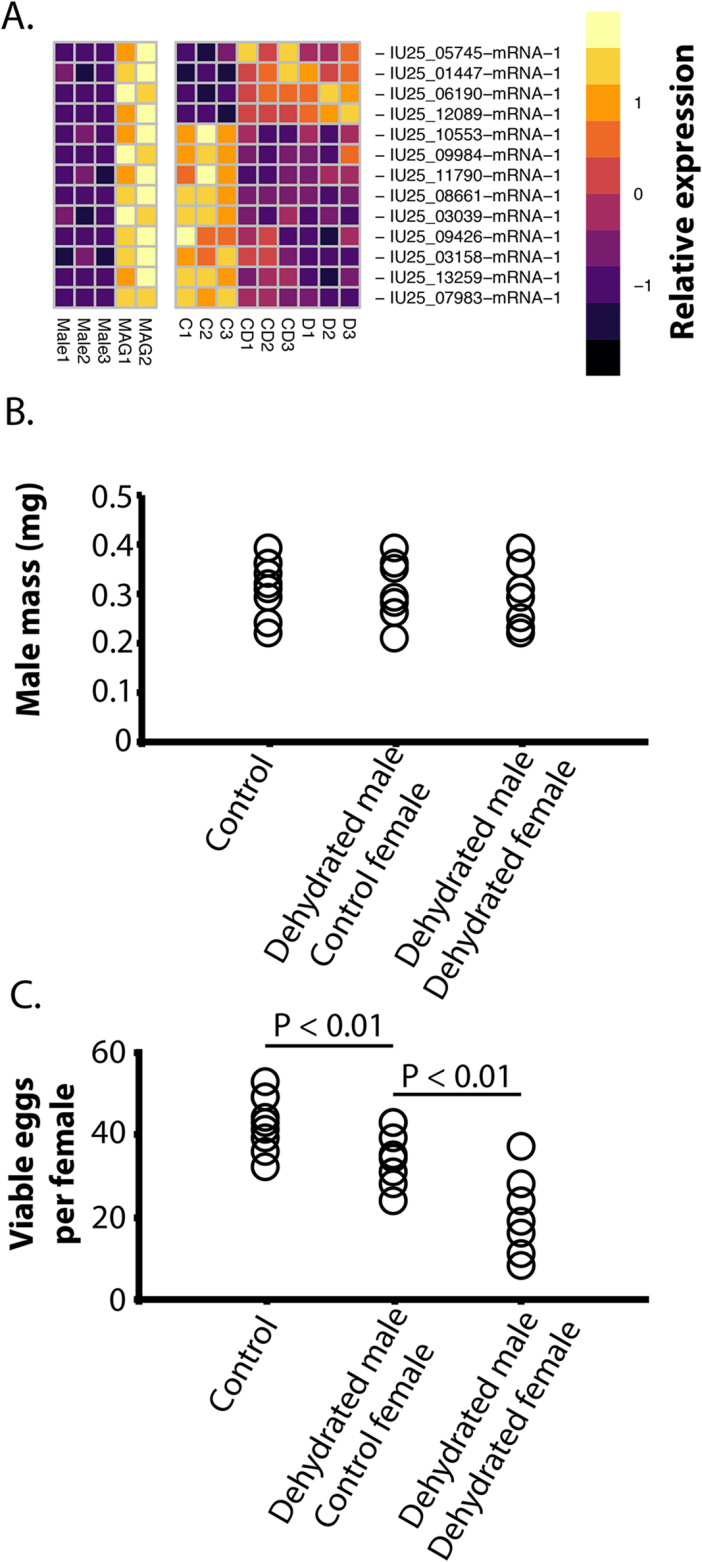
Impact of larval dehydration stress on male fertility. (**A**) Expression profiles of male-associated genes with expression differences after larval dehydration (D, dehydration, C, control, and CD, cryoprotective dehydration, MAG, male accessory gland). (**B**) Mass of males used in mating experiments from dehydrated or control larvae. (**C**) Viable eggs produced (control or dehydrated) following copulation with dehydrated or control males. Analysis of variance (ANOVA) was utilized to examine statistical differences with the use of R statistic packages. Tukey’s test was used as a post-hoc test to examine significance between each treatment. Bars above indicate significance at P < 0.05 unless otherwise noted.

We next examined the impact of larval dehydration stress on male fertility. To do so, we re-analyzed previously published RNA-seq data^62^ for larvae following dehydration or cryoprotective dehydration for differences in expression of transcripts enriched in male accessory glands (Fig. 11A). Thirteen genes enriched in male accessory glands were differentially expressed in the adult male following larval dehydration compared to fully-hydrated individuals (Fig. 11A). Enrichment of glutathione transferase activity and phosphatidylcholine metabolic processes were detected. Male body mass was not altered following dehydration stress, but mating was substantially compromised (Fig. 11B,C). When males dehydrated as larvae mated with unstressed females, fewer viable eggs were deposited, and additive reductions were noted if dehydrated males mated with females that were also dehydrated as larvae (Fig. 11C). Importantly, there was no reduction in total egg number between control and dehydrated males, but there was a reduction in total eggs produced when dehydrated males and females were mated (ANOVA: F_2,21_ = 5.11: P < 0.05, Tukey’s: P < 0.05). This reduction in eggs was not significantly more than when control males mated with dehydrated females (Fig. 10F, t-test, t-statistic_1,12_ = 1.14: P > 0.05),thus dehydrating males did not account for the total reduction in viable eggs. The combined effect is likely due to reduce egg output and viability. These results indicate an impact on male fertility brought about by dehydration stress during larval life.

### Role of gel as a nutrient source

**Figure 12:**
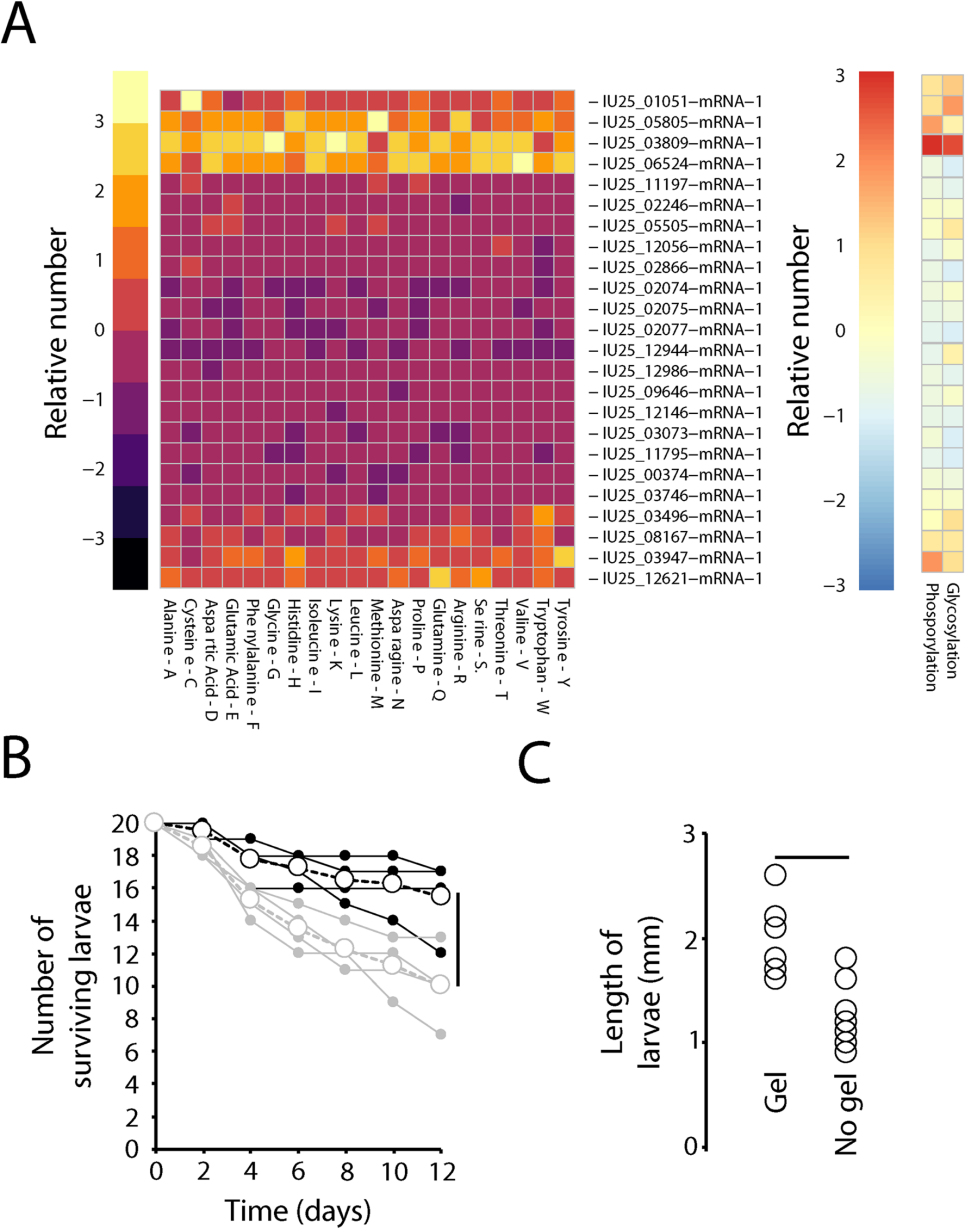
Accessory gland gel is critical for larval development. (**A**) Amino acid composition and putative phosphate and glycosylation sites of gel proteins determined based on Fig. 6 based on relative number associated with each predicted protein sequence. Gene identification is based on those used in the *B. antarctica* genome^57^. Relative amounts are based on comparison levels between columns. (**B**) Survival of developing larvae with (black) and without (gray) gel presence at larval ecdysis. Open circles are the average and filled circles are each replicate. (**C**) Larvae length after 20 days with and without gel at larval ecdysis. Bar indicates significance at P < 0.05. T-test was utilized to examine statistical differences with the use of R statistic packages. Bars above or beside indicate significance at P < 0.05.

Analyses of major gel protein components (over 3% of the total proteins of the gel) was performed. Amino acid content of the major protein components revealed that all essential amino acids are present within the gel (Fig. 12A). Predicted glycosylation and phosphorylation sites of the proteins suggest that these proteins could provide phosphate and sugar resources for the developing larva as they consume the gel (Fig. 12). Spectrophotometric analysis of the gel revealed that 81%, 14%, and 5% of the caloric content consisted of proteins, carbohydrates, and lipids, respectively. Larvae were denied access to the gel to determine its impact on larval growth (Fig. 12B). Fewer of the gel-less larvae were alive after 12 days (Fig. 12B, t-test: t-statistic_1,6_ = 2.81: P < 0.05), and, after 20 days, larvae that survived were nearly 30% smaller than larvae that hatched from eggs with free access to the gel (Fig. 12C, t-test: t-statistic_1,16_ = 2.42: P < 0.05). Importantly, these larvae were provided access to algae and other organic debris with and without the gel, indicating that non-gel food resources do not fully compensate for loss of the gel as an early food source. These results underscore the value of the gel for successful larval development.

### Thermal and dehydration buffering by the gel

We performed experiments to determine whether the gel impacts dehydration resistance in developing eggs. When gel-less eggs were held in desiccating conditions (75% relative humidity) at 4 °C for 12 h and subsequently treated with water, no viable eggs were detected, even when the eggs were subsequently covered with gel to promote growth (0% for all three replicates). By contrast, viability of eggs encased in gel was 81 ± 2% under the same conditions, suggesting that the accessory gel protects eggs against dehydration-induced mortality. These results indicate that the gel is likely critical for maintaining water homeostasis within the egg and possibly for the developing embryo as well.

**Figure 13:**
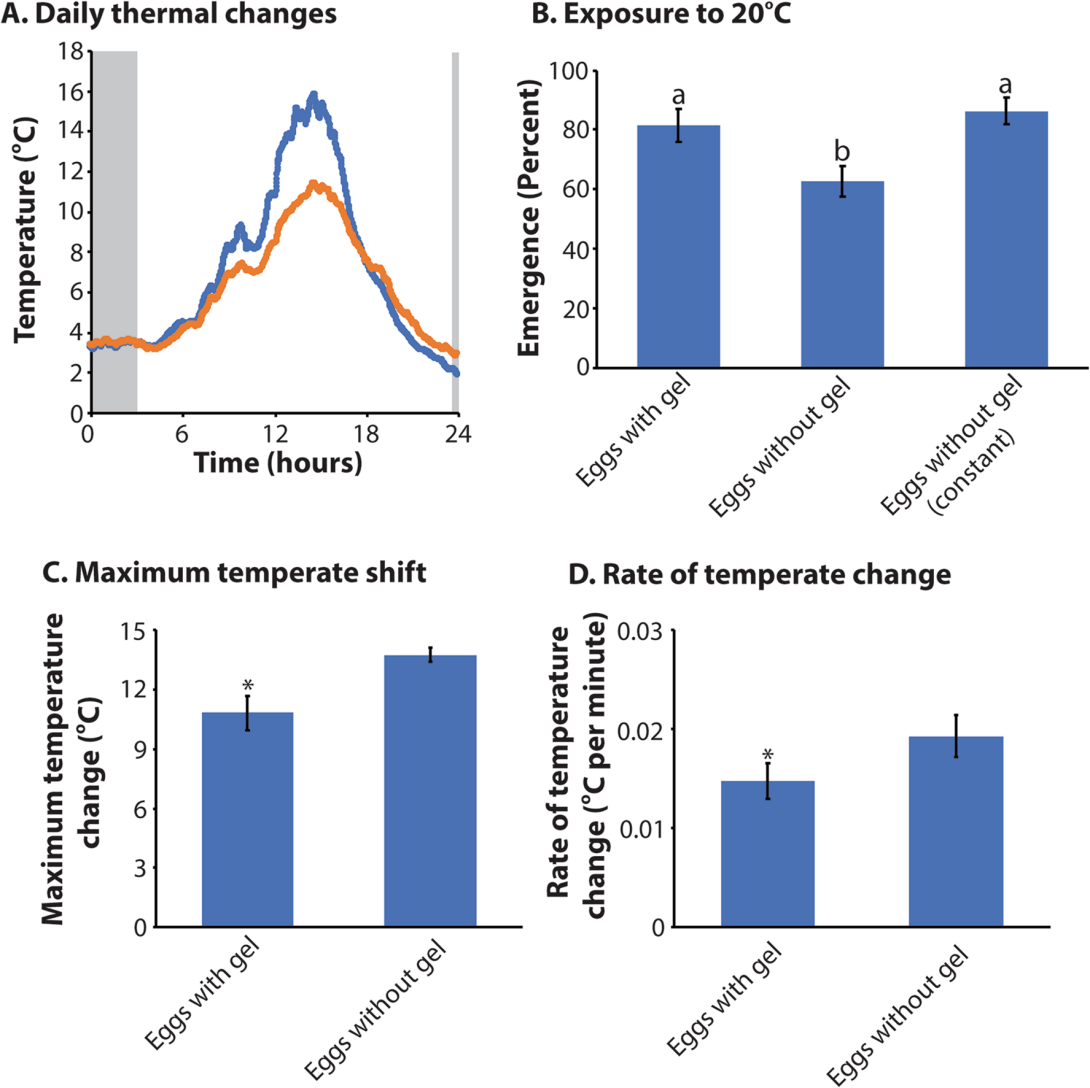
Role of accessory gland gel in relation to thermal buffering of eggs. (**A**) Representative thermal profile within (orange) and outside (blue) gel-egg mixture under field conditions from Cormorant Island. (**B**) Egg viability following exposure to 20 °C for 3 h. Eggs without gel (constant) were held at 4 °C for the duration of the trial. Maximum temperature change during 24 h period. N = 6 for each treatment. (**C**) Maximum temperature change during the course of a single day. (**D**) Rate of temperature change (minimum to maximum). T-test or Analysis of variance (ANOVA) was utilized to examine statistical differences with the use of R statistic packages. Tukey’s test was used as a post-hoc test to examine significant between each treatment. *or letters above indicates significance at P < 0.05 unless otherwise noted.

We also examined whether the gel acts as a thermal buffer. First, temperature changes were examined within the gel (next to the eggs) and on the surface immediately adjacent to the eggs (Fig. 13A), Gel-less eggs exposed to 20 °C displayed reduced viability when compared to eggs encased in gel and to those held constantly at 4 °C (Fig. 13B, ANOVA: F_2,19_ = 4.17: P < 0.05; Tukey’s: P < 0.05). The gel buffered temperature changes during the course of the day by reducing both the maximum temperature (t-test: t-statistic_1,10_ = 2.91: P < 0.05, Fig. 13C) and the rate of temperature change (t-test: t-statistic_1,10_ = 3.13: P < 0.05, Fig. 13D). These results indicate that the accessory gland gel provides both thermal and dehydration protection to the eggs.

### Population modeling

**Figure 14:**
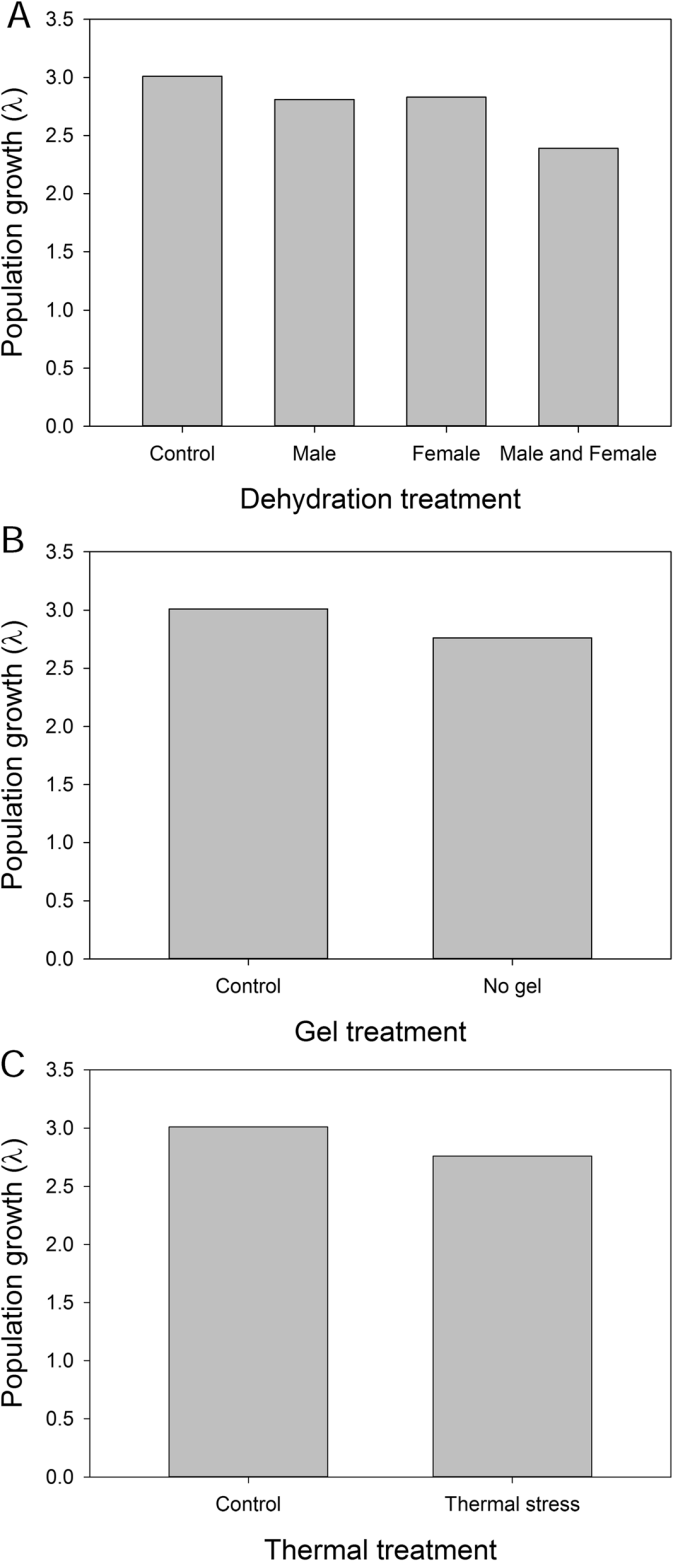
Population growth is impacted by dehydration and thermal stress in developing larvae. (**A**) Population growth following altered egg production due to dehydration exposure as larvae in males, females, and both sexes combined compared to control (no dehydration of larvae). (**B**) Growth based on the presence or absence of the gel under favorable conditions. (**C**) Impact of thermal stress on egg viability with and without accessory gland gel.

Dehydration of males, females, and both males and females together were examined to assess the impact of these stress on population growth relative to control populations (Fig. 14). The combined effect of male and female dehydration had the largest effect, reducing population growth by nearly 21%. Lack of gel also negatively impacted population growth, resulting in a 13% reduction relative to controls. The effect of thermal stress on egg viability would reduce population growth by 8%. In spite of these scenarios, populations would still be expected to increase in abundance under each specific stress. Under the “worst case scenario” where fecundity is reduced by male and female dehydration, larval survival is reduced by thermal stress, and there is a lack of gel reducing egg survival, population growth rate is negative (λ = 0.95), indicating declining population size. We note that all these growth rates are fairly liberal because of the relatively short time over which larval survival was observed (12 days) relative to the time spent as larvae in nature. In addition, it is likely that other factors, such as starvation, freezing, or pathogen attack, could occur, resulting in additive negative impacts on population growth. In contrast, multiple matings could improve female fertility.

## Discussion

**Figure 15:**
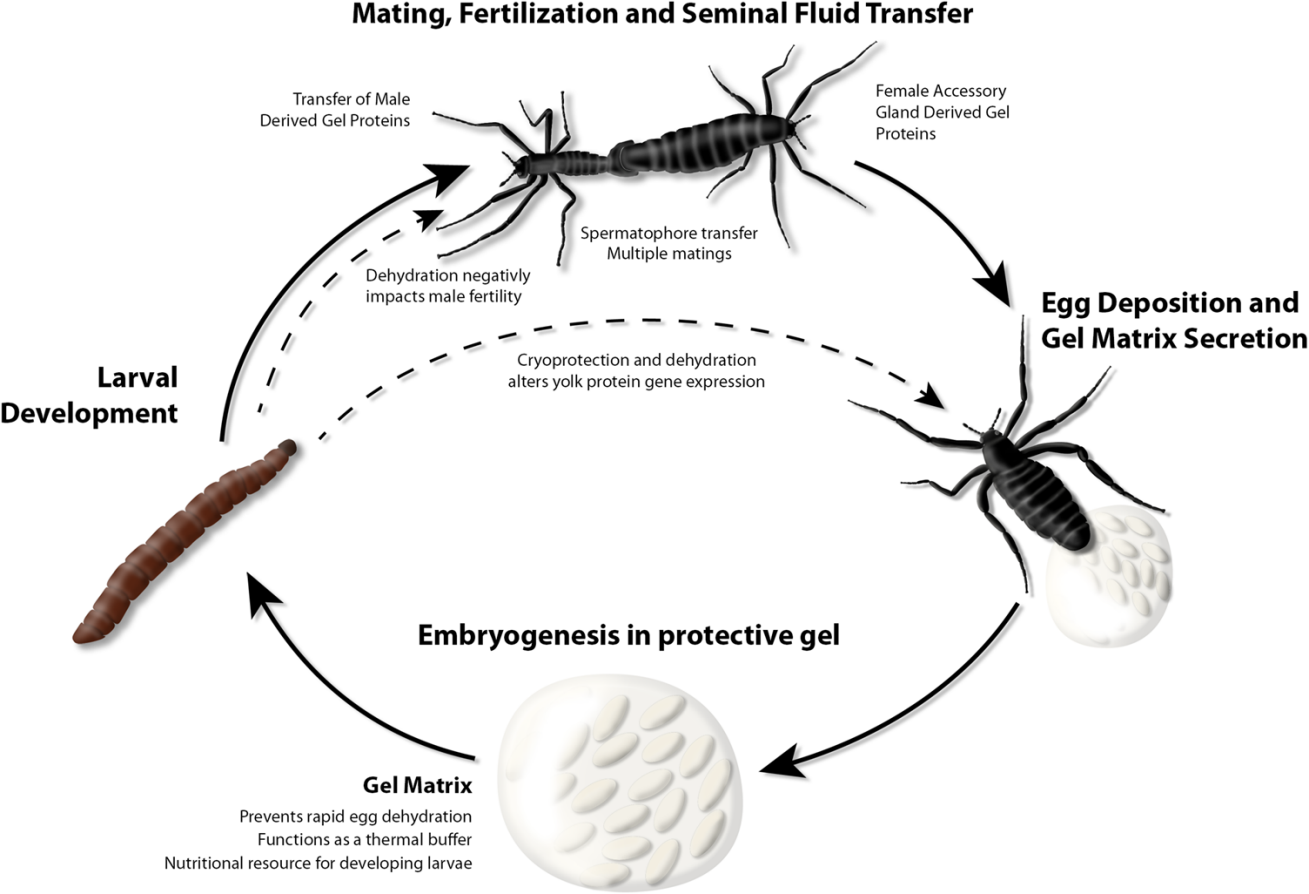
Summary of Antarctic midge reproduction. Larval development (four stages) is condensed into a single representation for all stages. Adults live approximately 2–3 weeks. Egg development occurs over 30 days. Impact of specific conditions are highlighted based on experimental evidence from this study.

This study examined reproductive biology of the Antarctic midge with the goal of establishing key molecular mechanisms associated with male and female biology. Combined RNA-seq and proteomics established the transcriptional components of reproduction and protein constituents deposited in the gel surrounding the egg. Specifically, we examined whether the gel that encases the eggs alters egg viability and larval survival and examined the impact of larval dehydration exposure on adult fertility. Little is known about midge reproductive biology and we have summarized the major findings of this study in Fig. 15. Thus, this study will hopefully provide a foundation for the fields of Antarctic and midge reproductive biology. Lastly, we modeled the impact of multiple reproductive factors on population growth, each of which exert minor effects but in combination could yield a negative growth rate. Our results highlight the importance of understanding the reproductive biology of this Antarctic insect, a species restricted to a limited geographic region and a specific habitat.

The large proportion of apparently conserved, female-specific or female-enriched genes that are uncharacterized (≈44%) suggests there are many aspects of *Belgica* female physiology that remain poorly understood. However, the results of the GO analysis of the female-enriched gene set are consistent with results reported for *Drosophila* and other insect systems, which are most likely associated with the development of oocytes^36,66–68^. In an analysis of genes differentially expressed between germline-ablated and gonadectomized females in *Drosophila*, Parisi et al. found enrichment in terms linked to metabolism^69^. Adult *Belgica* are not active eaters, thus the larva enriched gene set is replete with putative trypsins, chymotrypsin like proteins, and lipases^70–74^, which provide the necessary resources for subsequent oocyte development in adult females. Oogenic gene expression has also been linked to nutrient sensing in *Drosophila*, a feature we also note in *Belgica*^4,75,76^. Enrichment of terms in *B. antarctica* related to metabolism and RNA processing, including components of ribosomes as protein-building machinery, are most likely associated with oogenesis-related biosynthesis. This could include mobilizing lipids and proteins from the adult fat body during vitellogenesis, as well as synthesis of components of the egg gel excreted from the accessory gland. Transcription factor analyses revealed a subset of genes likely to have critical roles in female biology and thus warrant further, more directed studies. One identified transcription factor was *Drosophila* Mad which is a participant in the signaling pathway of decapentaplegic (dpp), a morphogenetic protein that plays a role in regulating the development of egg polarity and early embryonic development^77,78^.

Many enriched GO terms can also be tied to gametogenesis and embryogenesis or *Belgica* females. Terms associated with cell cycle control and DNA/RNA/chromatin handling are among the main examples. Beyond meiotic divisions that result in formation of gametes, which of course takes place in males as well, females may have to exercise more precise control over the timing of oocyte maturation, deploying cell-cycle modulators, transcription regulation machinery, and/or RNA silencing factors^79–81^. TF analyses revealed a NF-kappa relish-like transcription factor as a potential regulator of transcript levels for the female-enriched gene set. Indeed, NF-kappaB transcription factors play well-established roles in transcriptional regulation of cytokines, molecules involved in cell cycle control, and development^82–84^. Curiously, this TF has its highest expression in males despite a paucity of binding sites in the regulatory regions of the male-enriched gene set. However, expression in the male accessory gland is limited and lower than that of the female or female accessory gland. The meaning of these findings remains unclear and warrants additional targeted studies of this NF-kappa relish-like transcription factor.

The female also produces developmental factors that play a role in successful embryogenesis, such as mRNAs transferred into the oocyte, hormone biosynthesis precursors, and other general cytoplasmic components (sperm are quite cytoplasm-poor relative to eggs)^85^. Among the most highly enriched genes in females relative to males (> 1000-fold higher) are a handful of genes known to be involved in embryonic patterning in insects, such as *nanos*, *oskar*, and multiple innexins, components of invertebrate gap junctions involved in intercellular communication during embryogenesis^86–90^. This lends support to the notion that the female-enriched gene set is comprised largely of genes regulating and supporting oogenesis and embryogenesis. In addition, paternal DNA must be de-compacted after fertilization, meaning that sperm nucleotide binding proteins must be degraded^91^. The enzymes/proteins that participate in this process are likely already present in the egg, and therefore maternally contributed^92^. This is followed by histone/chromatin assembly which is dependent on maternally provided histones and nucleosome assembly factors^92,93^. Male-contributed mitochondrial DNA must also be degraded^94,95^. This further accounts for the prevalence of terms associated with histones and chromosome organization. Finally, in *Drosophila,* nurse cells surrounding the oocyte are known to polyploidize regions of their nuclear DNA to enhance transcription and support provisioning of the oocyte^96–98^. This provides yet another physiological link to DNA replication and chromosome organization.

The dominant GO terms connected to the male-enriched gene set are ‘anion transport’ and ‘carboxylic acid biosynthesis.’ These are both terms that can be linked to spermatogenesis and may also be related to the differing metabolic needs of males, females, and larvae: larvae eat, digest, and grow; females conduct vitellogenesis, oogenesis, and embryogenesis; and males likely devote the bulk of their energy store to spermatogenesis and mating^99^. The two terms may be interrelated. For example, genes involved in synthesis and transport of pyruvate, a carboxylate anion and the starting substrate of the tricarboxylic acid (TCA) cycle, could be associated with either GO term. The specific functional role of pyruvate metabolism in sperm viability has yet to be established, but mitochondrial activity is critical for fertility and a mitochondrial pyruvate transporter that is uniquely expressed in the male germline has been identified in placental mammals^100^. However, inhibition of pyruvate transporters in the mitochondrial membrane does not appear to affect sperm motility, lending support to the notion that sperm motility is fueled primarily by glycolysis (not import of pyruvate into mitochondria)^100^. An alternative role is that pyruvate metabolism may play a role in viability, as it has been associated with potential sperm viability in *An. gambiae*^26^. TCA-based energy production must play a role in some other aspect of sperm viability^100^. The fact that a mitochondrial pyruvate transporter is important in sperm viability suggests that pyruvate transport and metabolism contribute to the significance of the GO terms ‘tricarboxylic acid cycle,’ ‘monocarboxylic acid metabolic process,’ and ‘anion transport.’ Additionally, sperm are thought to take up lactate and pyruvate through general monocarboxylic acid transporters (MCTs) in humans^100–102^. Perhaps there is an analogous uptake of energetically important organic acids in the seminiferous tubules of *Belgica* and other insects. Finally, pyruvate may be useful in supplying Acetyl-CoA for the histone acetylation that is essential for chromatin condensation during spermatogenesis^100–102^.

Overall, males showed far fewer genes with enriched expression relative to the female. Of the 12 genes that are > 1000-fold higher in the male compared to other stages, three are putative metalloendopeptidases. These may be involved in the proteolytic processing of amyloid precursor proteins (APPs), which are integral components of sperm membranes in humans, though their specific roles have not been determined^103^. Also of interest in this small set of highly enriched genes are a few genes commonly, though not exclusively, associated with immunity: a leucine rich immune protein TM, a toll protein, and an apparent homolog of the transcription factor NF-X1^104–107^. This transcription factor binds X-box motifs to regulate many eukaryotic genes and is generally thought to be a negative regulator of transcription^108^. The homolog of this transcription factor identified in *D. melanogaster*, named shuttlecraft (stc), is essential for normal embryonic development and is expressed most highly in the embryo CNS. Moreover, expression of this gene is highest in the *Drosophila* ovary, and the promoters of many maternally contributed genes known to be crucial in embryonic development contain X-box motifs, such as oskar*,* torpedo*,* pumilio*,* and vitelline membrane proteins^108^. Since this *Belgica* transcription factor NF-X1 has transcript levels roughly 24-fold higher in the male accessory gland than in the whole carcass, this TF likely plays a critical role in the generation of male accessory gland products.

Gene ontology analysis of the larvae-enriched gene set revealed a preponderance of terms related to peptidases, hydrolases, and detoxification activity. These GO terms are related to digestion and detoxification of ingested materials by the growing larvae—neither of which would be relevant issues for the non-feeding adults. One cytochrome p450 (CYP6Z2), up-regulated in larvae, has been implicated in chemical resistance in *Anopheles* mosquitoes^109^ and therefore may be critical for larval survival in a potentially toxic microhabitat, such as seabird guano^110^.

Besides the ingestion of food, larvae of *B. antarctica* are also longer-lived and must face the seasonal challenges of permanent residence in Antarctica^2,5^. In particular, larvae must survive freezing and desiccation during the austral winter along with potential thermal stress during summer^7,8,10,111–113^. Genes enhancing survival under these conditions, such as heat shock proteins^7,8,10,111–113^, are up-regulated in larvae. Based on previous studies in *Belgica* and other species that are desiccation and freeze tolerant, genes of importance could include oxido-reductase related enzymes (e.g. cytochrome p450s), protein repair methyltransferases, hemoglobins, aquaporins, and enzymes involved in trehalose metabolism^7,8,10,111–117^. Many of these categories are indeed enriched in the larvae and are likely substantial factors in the stress resistance abilities of *B*. *antarctica*. Note that the presence of hemoglobin in insect hemolymph is unique to chironomids^118–120^ and furthermore it is thought to be unique to the larval stage. This could partially explain the prevalence of iron binding as a GO term enriched in the larvae and conserved among the midge species analyzed^118–120^.

Most prevalent among GO terms enriched in the larval gene set are those associated with aminoglycan metabolism. This set includes, for example, multiple putative N-acetylgalactosaminyltransferases, which catalyze the initial glycosylation of serine and threonine residues^121^. Mucin type O-glycosylation occurs commonly on proteins with extracellular domains, comprising a portion of the extracellular matrix, and it is thought to be important in intercellular communication and adhesion^121^. In *Drosophila*, enzymes responsible for building glycosylated proteins, such as mucins, are critical for embryonic development, particularly for the CNS^121–123^. Maternal and zygotic O-linked glycans have also been implicated in proper respiratory development. These O-glycans are termed *pgant* in *Drosophila*^121–123^. One essential gene, *pgant4*, is involved in regulating gut acidification, but this is not the only such gene required in the digestive system^121,124^. The larva enriched gene set in *Belgica* includes putative homologs of *pgant4* and *pgant6*, as well as the essential genes CG30463 and C1GalTA, both involved in mucin type O-linked glycosylation. However, larva-enriched GO terms, as mentioned above, are dominated by hydrolysis and catabolic processes, including glycosaminoglycan catabolism. This may be a sign of the breakdown of extracellular materials associated with growth and development or an indication of a diet rich in aminoglycans.

Composition of male accessory glands has been a major focus of research in numerous insects. These products influence fertilization rates, subsequent female receptivity to courtship, and are critical in the composition of the spermatophore^19–21,26–29,33,37–39,125^. Many of the male accessory gland genes from *Belgica* have orthologs based on predicted genes from midge and mosquito genomes, but few overlapping orthologs were identified that are expressed in the male accessory gland of *B. antarctica* compared to those expressed in the male reproductive tissues of mosquitoes^25,26^. This is not surprising since a similar lack in orthology among male accessory gland products has been observed between *Drosophila* and *Glossina* and other higher flies^34,36^. We did not conduct biological examination of specific roles for accessory gland proteins from *B. antarctica*, such as whether their transfer during mating impacts refractoriness of females, as noted in many species^19,21,125,126^. There is an enrichment for genes associated with glycoprotein synthase, which is unsurprising as glycoproteins are common constituents of seminal fluid^21,51^. In addition, there were specific serine proteases, immune factors, and products involved in response to oxidative stress. These are similar to those observed in other fly species^19,21,125–127^ and likely serve to preserve sperm viability but could impact aspects such as female biology^128,129^. These factors are likely critical to male success, as female midges will mate on multiple occasions. It is important to note that females will deposit eggs with and without fertilization^11^, thus suggesting that factors supplied in the spermatophore are not essential for ovulation and oviposition as in some other fly species^19,21,125,126^.

Many, but not all, products present within the gel encasing the eggs were expressed in the female’s accessory gland. A comparison of our results to expression pattern in mosquito female tracts^25^ revealed few genes with overlapping expression^25^. Female accessory gland studies in dipteran and other insect systems have been limited. Even in *Drosophila*, the female accessory gland (paraovaria) remains one of the most understudied organs^130^. In tsetse flies, this organ provides nourishment to developing intrauterine larvae^22,36,131^, but there are no similarities, beyond standard housekeeping genes, between this organ in tsetse flies and *B. antarctica*. Of interest, regulation of transcript expression within the female accessory gland seems to be conserved^130^, suggesting that regulatory aspects may be similar but drive expression of specific genes related to unique functions of this organ in different flies. Transcription factor analyses identified a single gene (IU25_08656) that has enriched levels of both binding sites upstream of female accessory gland enriched genes and is itself expressed in the female accessory gland. This is an uncharacterized zinc finger protein, but is likely to have a critical role in female reproductive function for *B*. *antarctica*.

The three major protein components of the gel surrounding the eggs are vitellogenin, larval serum protein, and apolipophorin, all which are reasonable components of a gel whose primary function includes fueling the development of larvae upon hatching. *Belgica* larvae exhibit “drinking behavior” shortly before hatching, suggesting that up to this point embryonic development is fueled by nutrient reserves present in the egg at the time of oviposition^11^. Upon hatching, the gel is ingested by the larva, making it the first meal fueling their development. Larval serum protein likely acts as an amino acid reservoir, while vitellogenin and apolipoproteins could provide sources for fatty acids, carbohydrates, and amino acid reserves. The gel may also contribute other elements in smaller amounts, such as pre-synthesized developmental hormones in an inactive form, hormone precursors, proenzymes, and enzyme cofactors that are important for continued development^11^. Larval serum protein is a storage protein belonging to the family of hexamerins^63–65^. Genes for such proteins are often highly expressed in the final larval instar preceding pupation^63–65^ and serve as a nutrient reserve for developing pupae and newly emerged adult. During the sweeping morphological changes that accompany metamorphosis in holometabolous insects like *Belgica*, such storage proteins are extracted from the larval fat body, transferred into the hemolymph, and subsequently re-sequestered in the newly formed adult fat body^63–65,132,133^. The following scenario is the likely fate of the larval serum protein found in the egg gel: Initially re-sequestered by the adult fat body and shuttled to the female accessory gland with vitellogenin, or possibly directly allocated to the female accessory gland during adult development without a layover in the fat body. Importantly, lack of expression in the female adult indicates these proteins must be synthesized in the larva for subsequent use by the adult.

Along with establishing components of the female accessory gland derived gel, a major goal of this study was to identify functional roles of this gel and to determine if the gel components are impacted by stress in the developing larvae. Based on our results, one of the main functions of the gel is to provide a nutritional resource, which leads to higher larval survival. Products of the accessory glands of females have been documented as food sources for insect species among many orders^22,36,53,134,135^. Production of gel substances surrounding eggs have been noted in other species, such as in stink bugs, where the gel serves as a source of nutrition, protects the eggs, and provides a vehicle to transfer microbial symbionts^53^. Similar to our study, removal of the gel inhibits juvenile development in stink bugs, but in contrast, removal of the gel around the stink bug eggs has little impact on hatching success. In addition to providing a critical nutritional source and preventing dehydration, we show that the egg gel acts as a thermal buffer that limits temperature extremes. Our de novo assembly of the female accessory gland failed to detect signatures of microbial symbionts within the gel, indicating that the gel does not serve as a mechanism of bacterial transfer to the developing larvae. This lack of microbial presence could very well limit microbial exposure of the midge until after larvae emerge from the gel. Accessory gland products also have been demonstrated to possess antimicrobial properties^21,136,137^; this is a possibility for *B. antarctica* because putative immune peptides are present in the gel.

Larval dehydration stress had a major impact on fertility of both adult males and females. The most likely cause of reduced fecundity in males and females is a direct reduction in larval serum protein, a hexamerin that acts as a storage protein in larvae^63–65^. This hexamerin represents one of the highest expressed transcripts in developing larvae, so its accumulation during the juvenile stages is likely a critical source for amino acids and energy reserves that are critical for both production of eggs and generation of accessory gland components of both males and females. This effect is likely even more pronounced in *B. antarctica* because adults do not feed or even readily drink water^138^, thus they rely solely on nutrients obtained during the larval stage for both somatotropic and gonadotropic development and maintenance. Along with acting as a nutritional source, the larval serum protein generated in juvenile stage of females is likely incorporated into the accessory gland gel, suggesting that a reduction in this product may directly impact gel composition. Larval nutritional status has a direct impact on fecundity in numerous insect systems^139–141^, including other midges^16^. To the best of our knowledge, larval dehydration has not previously been examined in relation to subsequent adult fecundity. Other stressful conditions, such as chemical exposure, have impaired both larval development and subsequent adult reproduction in a non-biting midge, *Chironomus riparius*^17^.

This study provides an encompassing view of reproductive biology of the Antarctic midge, from molecular mechanisms to the impact of larval stress exposure on adult fecundity. Key findings are summarized in Fig. 15. This is followed by population growth modeling to establish how these factors directly impact persistence of this insect in its limited Antarctic range. Population modeling revealed that each factor (dehydration stress, lack of gel, thermal stress), by itself, has a small impact on population growth but combined factors likely result in negative population growth. The limited reproductive window of 2–3 weeks makes understanding both male and female reproduction critical for understanding how this midge survives in Antarctica. Studies on the reproductive biology of flies have been limited largely to *Drosophila* and disease vectors (sand flies, mosquitoes, etc.), and our results expand into Chironomidae to provide the groundwork for future studies with this dipteran system.

## Materials and methods

### Midge collections

Antarctic midges were collected from islands near Palmer Station (64° 46′ S, 64° 04′ W) in January 2007 and January 2017. Males and females were separated based on major morphological characters described previously^1,2^, homogenized at the research station, and stored in Trizol at − 70 °C for shipment to the University of Cincinnati. Female and male accessory glands were also dissected (N = 20–30 per replicate) and stored in Trizol (Invitrogen) similar to whole body stages.

Larvae were collected from the same location as adults. Larvae embedded in organic debris were returned to Palmer Station and extracted into ice water with a modified Berlese funnel. Following recovery, larvae were stored with substrate from their natural habitat (rocks, soils, moss, and the alga *Prasiola crispa*, which serves as a food source for *B. antarctica*) at 2–4 °C. Larvae were shipped to the University of Cincinnati and stored under similar conditions until they were used in studies examining the impact of larval stress or gel presence on adult fertility or egg viability, respectively.

### RNA extraction and processing

RNA was extracted from the midges by homogenization (BeadBlaster 24, Benchmark Scientific) in Trizol reagent (Invitrogen), using manufacturer’s protocols with slight modification based on other studies of invertebrates^58,142^. Extracted RNA was treated with DNase I (Thermo Scientific) and cleaned with a GeneJet RNA Cleanup and Concentration Micro Kit (Thermo Scientific) according to manufacturer’s protocols. RNA concentration and quality were examined with a NanoDrop 2000 (Thermo Scientific). For RNA-seq, three biological replicates were prepared from the following sample: males, females, females after oviposition, and female accessory glands. Three replicates were prepared from the male accessory glands, but only two were sequenced due to low quality RNA in the third sample.

Poly(A) libraries were prepared by the DNA Sequencing and Genotyping Core at the Cincinnati Children’s Hospital Medical Center. RNA was quantified using a Qubit 3.0 Fluorometer (Life Technologies). Total RNA (150–300 ng) was poly(A) selected and reverse transcribed using a TruSeq Stranded mRNA Library Preparation Kit (Illumina). An 8-base molecular barcode was added to allow for multiplexing and, following 15 cycles of PCR amplification, each library was sequenced on a HiSeq 2500 sequencing system (Illumina) in Rapid Mode. For each sample, 30–40 million paired-end reads at 75 bases in length were generated. Raw RNA-seq data have been deposited at the National Center for Biotechnology Information (NCBI) Sequence Read Archive: Bio-project PRJNA576639. Along with the RNA-seq samples collected for this study, larval (control, dehydration, and cryoprotective dehydration) samples were acquired from Teets et al.^62^ under the NCBI Bioproject PRJNA174315.

RNA-seq reads were trimmed for quality (Phred score limit of 0.05) and sequences with ambiguities were removed. In addition, five and eight nucleotides were removed from the 5′ and 3′ ends, respectively, and sequences shorter than 45 bases were removed. Reads before and after cleaning and trimming were examined with FastQC for quality (S. Andrews https://www.bioinformatics.babraham.ac.uk/projects/fastqc) to verify quality of each set.

### Gene expression analyses

RNA-seq analyses were conducted using two distinct pipelines. The first method utilized was CLC Genomics (Qiagen), as previously described^58,142^. Briefly, reads were mapped to contigs with a cutoff of at least 80% of the read matching at 90% identity with a mismatch cost of 2. Each read was permitted to align to only 20 contigs. Expression values were based on total read counts in each sample calculated as transcripts per million reads mapped. EdgeR was used for statistical analysis of the resulting alignments. A multiple comparison correction was performed (false discovery rate, FDR). Genes were considered to be differentially expressed if the fold change was greater than 2.0 and the P-value was < 0.05. In addition, genes were required to have at least 5 mapped reads per sample to be retained for further analyses. For whole-carcass expression analyses, genes were considered sample-specific if enrichment was noted in relation to both of the other whole-carcass datasets. For accessory gland specific analyses, genes were considered tissue specific if they were enriched relative to the relevant whole-carcass dataset (female or male). In addition, a de novo assembly was conducted on the female accessory gland RNA-seq datasets using Trinity^143^ based on standard methods to determine if bacterial symbionts were present in this organ and could be transferred to the egg while in the gel. This was accomplished by BLASTx comparison to predicted gene sets from *B. antarctica*, *D. melanogaster*, and *A. gambiae* and the *B. antarctica* genome. This was followed by comparison to the NCBI NR database for bacterial sequences. Sequences that had no matches or only matched bacteria were further compared to the entire NCBI NR database and expression levels were assessed as described above.

The second method for examining transcript expression involved utilization of RNA-seq tools available through the Galaxy software package (www.https://usegalaxy.org/)^144,145^ using Salmon with the suggested default settings. Differential gene expression analysis was performed using the DeSeq2 package^146^. A general linearized model assuming a binomial distribution followed by a false discovery rate (FDR) approach were utilized to account for multiple testing. Cut-off values for significance, enrichment and sample-specificity were the same as those used in analysis conducted with CLC Genomics.

Transcripts identified as sex- or development-specific were examined using the CLC-based pipeline as there was over 95% overlap between each RNA-seq analysis method. Pathways enriched within males, females, and larvae were identified with a combination of Database for Annotation, Visualization and Integrated Discovery (DAVID^147^), Blast2GO enrichment analyses^148^, CLC gene set enrichment analysis^149^, and g:Profiler^59^. Due to the taxonomic limitations of DAVID, sets of enriched transcripts were compared by BLASTx to the *An. gambiae* and to the *D*. *melanogaster* RefSeq protein datasets to identify homologous sequences. Blast hits (e-value < 0.001) from these two species were submitted to DAVID. There was considerable overlap between the results, and only the CLC-based methods were used in subsequent analyses.

Lastly, we utilized weighted correlation network analysis (WGCNA) to identify specific modules of genes that have similar expression profiles across larvae and adult stages along with accessory glands^60^. WGCNA was used to construct correlation networks and describe correlation between gene expression across samples in RNA-seq or microarray data. Genes sharing similar patterns of expression across samples were clustered into modules to identify groups of biologically significant genes that were particular to one of the sample groups. For this analysis, RNA-seq data were screened for genes of zero variance prior to WGCNA, leaving 13,424 genes for signed network construction. The minimum module size allowed was 20 and the soft power was set to 14 as determined by the package’s scale-free topology function. Modules exhibiting the highest Pearson correlation coefficient were selected for further analysis to determine function and relationship to larvae, adults, or accessory glands. Modules identified as enriched in larvae, adults, or tissues were examined for enriched GO categories with the use of g:Prolifer and DAVID, as described earlier.

### Comparative analyses with other chironomid midges and *Anopheles* mosquitoes

This is the first study to examine genome-wide, sex- and stage-specific expression in a midge. However, a recent study examines sex-specific expression in four species of anopheline mosquitoes, a clade not distantly related to midges, along with expression specific to the male and female reproductive tracts (MRT and FRT, respectively^25^). The species covered in this study were *An. gambiae, An. minimus, An. albimanus,* and *An. arabiensis.* In addition, male accessory glands enriched genes for *B. antarctica* were directly compared to those from Anopheles male accessory glands^26^. Predicted gene sets from this study with stage and organs enriched expression were used in comparative analyses with *B. antarctica.* The genomes of four species of chironomid midges were also acquired: *Clunio* marinus^120^*, Parochlus steinenii*^150^*, Polypedilum vanderplanki*^119^*,* and *Polypedilum nubifer*^119^*.* Studies that resulted in these sequenced midge genomes did not include analyses of differential expression between sexes.

For comparative analyses between midges, predicted gene sets for *B. antarctica* were compared with genomes of each midge species, and results of these four analyses were pooled to establish putative sets of genes common to all five species. This analysis also resulted in identification of differentially expressed genes unique to *B. antarctica*. Similarly, predicted gene sets from *B. antarctica* were compared with each species of *Anopheles* mosquito. Antarctic midge gene sets were compared to *Anopheles* whole carcass gene sets, as well as reproductive tract-specific gene sets^25^. Lastly, we compared the expression of genes within the male accessory glands to orthologous gene sets that are uniformly expressed in the male accessory glands of mosquitoes^26^. The common gene sets produced by these analyses were then subjected to ontological analyses, using gProfiler, to establish sex-specific enriched pathways. tBLASTp analyses (e-value < 0.001) were performed using CLC Genomics Workbench (CLC bio Qiagen). Protein sequences were defined as orthologs if they were reciprocal-best BLASTp hits having an e-value < 10^−10^. Overlap was compared between these analyses to produce putative sex-specific transcript sets.

Transcription factors (TFs) and their predicted DNA binding motifs were identified based on methods used for other invertebrate genomes^36,68,151^. In brief, putative TFs were identified by scanning the amino acid sequences of all proteins for putative DNA binding domains using the HMMER software package^152^ and a compilation of Pfam DNA binding domain models^153^. Experimentally determined DNA binding motifs were then inferred from other species (*e.g., Drosophila*) based on amino acid identity, using previously established rules^154^. Using this collection of inferred DNA binding motifs, we examined enrichment of each motif within the 500 and 2000 bp promoter regions of genes demonstrating increased expression in a sex, stage or tissue specific manner. Specifically, we used the HOMER software package to calculate the enrichment of each motif, using the hypergeometric tests implemented in HOMER^155^. These results were then compared to the expression profiles of each TF to determine specific TF candidates that might regulate sex and reproduction associated genes.

### PCR and qPCR analyses

Select highly enriched genes of interest from males and females were verified by PCR and qPCR. Total RNA was extracted from males, females, larvae, female accessory glands, and male accessory glands as described in the RNA-seq section. The RNA was used as a template for cDNA synthesis using Superscript III reverse transcriptase according to the manufacturer’s protocols (Invitrogen). PCR was performed with gene-specific primer pairs (Table S15) using a DNA polymerase kit (Promega). The PCR conditions were 95 °C for 3 min, 35 cycles of 30 s at 95 °C, 52–56 °C for 1 min, and 1 min at 70 °C using an Eppendorf Mastercycler Pro Series. Three independent (biological) replicates were conducted for each sex or tissue stage.

qPCR analyses were conducted based on previously developed methods^156^. RNA was extracted as described previously from independent biological replicates for sexes and larvae. Complementary DNA (cDNA) was generated with a DyNAmo cDNA Synthesis Kit (Thermo Scientific). Each reaction used 250 ng RNA, 50 ng oligo (dT) primers, reaction buffer containing dNTPs and 5 mmol l^−1^ MgCl_2_, and M-MuLV RNase H + reverse transcriptase. KiCqStart SYBR Green qPCR ReadyMix (Sigma Aldrich, St Louis, MO, USA) along with 300 nmol l^−1^ forward and reverse primers, cDNA diluted 1:20, and nuclease-free water were used for all reactions. Primers were designed using Primer3 based on contigs obtained from the transcriptome analysis (Table S15). qPCR reactions were conducted using an Illumina Eco quantitative PCR system. Reactions were run according to previous studies^156^. Four biological replicates were examined for each sex, and three biological replicates were examined for each accessory gland. Expression levels were normalized to *rpl19* using the ΔΔCq method as previously described^156,157^. Fold change in gene expression was compared between larvae, males, females, and accessory glands followed by determination of the Pearson correlation coefficient (r) between the qPCR and Illumina data.

### Proteomics and nutritional analysis of accessory gland-derived gel

Samples were analyzed at the Proteomics and Metabolomics Laboratory at the University of Cincinnati. Two proteomic samples were collected by removing eggs from the gel with a micropipette and dissolving the gel in 1 × PBS with 0.1% Tween (6 eggs per sample). Samples (4 µg) were run on a 1D SDS PAGE gel and silver stained to confirm the presence of proteins; at least 15 distinct proteins could be visualized. Based on this initial characterization, gel proteins (6 µg) were run 2 cm into a 1D 4–12% Bis–Tris Invitrogen NuPage gel using MOPS buffer. Lanes were excised, reduced with 10 mM dithiothreitol, alkylated with Iodoacetamide and digested with trypsin according to standard protocol^157,158^. The resulting peptides were concentrated with a speed vac centrifuge and resuspended in 0.1% formic acid. Each sample (2 µg) was used in subsequent analyses. Nanoscale LC-electrospray ionization-MS/MS (nanoLC-ESI–MS/MS) analyses were performed on a TripleTOF 5600 (Sciex, Toronto, ON, Canada) coupled to an Eksigent (Dublin, CA) nanoLC ultra nanoflow system. Protein from each gel sample was loaded and analyzed as described^158,159^. The data were recorded using Analyst-TF (v.1.6) software and searched against the *B. antarctica* genome^57^ using the Protein Pilot program (Sciex). Gel proteins were compared to those with differential expression in specific tissues from our RNA-seq studies. Protein, carbohydrate, and lipid content were examined through spectrophotometric assays based upon methods described in Rosendale et al.^58^.

### Thermal buffering by the accessory gland gel

The gel has been suggested to serve as a source of nutrients for newly emerged larvae^1,11,160^. In addition to this role, we examined whether the gel increases thermal buffering capabilities of the egg compared to eggs directly deposited on the local substrate. To examine the effect, we placed an Omega thermocouple within six gels and immediately adjacent to these six gels at a field location near Palmer Station. Temperature was measured every minute over the course of 3 days.

To establish whether the gel prevents egg death caused by thermal stress, eggs with and without gels were exposed to 20 °C for 3 h. Females were allowed to lay their eggs onto moist filter paper disks (Whatman) which were placed in 50 ml centrifuge tubes before being transferred to a 4 °C water bath. Temperature was then ramped up to 20 °C over the course of four hours, 4 °C per hour, before slowly being reduced back to 4 °C over the same time course. These samples were compared to those that were held continuously at 4 °C without the gel. Following treatment, all eggs were maintained at 4 °C and monitored for larval emergence. Six egg masses were used for each treatment.

### Role of accessory gland gel in dehydration

To determine if the accessory gland gel could prevent egg dehydration, we subjected midge eggs to dehydrating conditions with and without the presence of the gel. All eggs were removed from the gel with a fine metal probe and half were carefully reinserted into the gel. Three groups of eggs with or without the gel were moved to 75% RH at 4 °C for 12 h. Following this treatment, eggs with no gel were placed back into the gel. Viability was determined by counting the number of larvae that emerged from the total number of eggs.

### Impact of larval dehydration stress on fecundity

To determine if larval dehydration stress impacts adult fecundity, we performed a re-analysis of published RNA-seq datasets that examined exposure of larvae to both standard and cryoprotective dehydration stress^62^. The previous RNA-seq study^62^ compared larvae that were quickly dehydrated (30% water loss) to those that had undergone a slower form of dehydration, cryoprotective dehydration (30% water loss). The resulting data sets were then examined to find genes with increased expression in male or female accessory glands.

To determine whether mating is directly impacted by dehydration stress, groups of 100 fourth instar larvae (final larval instar) treated as four replicates of 25 individuals were held at 75% RH until they lost 40% of their water content, based on mass determination of a small subset. Following dehydration, larvae were returned to standard rearing conditions and monitored every 12 h for the presence of pupae or newly emerged adults. Emergence incidences for adults was 90% across all treatments. Each adult was removed and stored separately at 98% RH, 4 °C until mating, which occurred no later than 4 days after treatment. The following mating pairs were examined: males from dehydrated larvae vs. control females, females from dehydrated larvae vs. control males, and both males and females from dehydrated larvae (6–8 mating pairs for each group). This low number of mating pairs examined was due to the fact there was significant asynchrony in adult emergence, offering a limited window when the short-lived adults from each treatments could be mated successfully. Males and females that failed to copulate were removed from the experiment. Viable eggs were determined by monitoring eggs until larval development was noted^11^. Mass changes were determined with a CAHN electrobalance and were based on wet masses of the individuals.

### Statistics

Replicates were independent biological samples. Sample sizes are listed in each method section or the figure legend. Percentage data were angularly transformed before analysis. Significance is indicated within each figure and/or in the figure legend. Statistical tests are listed within the respective section in the methods or in the figure legends. All statistical analyses were performed using JMP version 11 (SAS) or R-based packages. Heat maps were generated with pheatmap in the R environment.

### Population level effects

To explore population level effects of dehydration, gel surrounding the egg mass, and thermal stress we used a Leslie matrix approach^161^. Here, the dominant eigenvalue of the matrix is the population growth rate (λ). We simplified life history to egg to larvae to adult despite there being four larval instars, potentially occurring over several years. For control populations we used a mean fecundity of 42.9 (eggs laid), an egg survival rate of 0.82, and larval survival rate of 0.78. To determine effects of larval dehydration on population growth, we used fecundities (eggs laid) of 34.6, 35.6, and 21.5 for male, female, and male and female dehydration, respectively; egg and larval survivorship were assumed to be the same as control populations. To determine effects of the gel on population growth we used all parameters (as for control populations), but reduced larval survival to 0.5, based on the experimental data from our studies. Similarly, to investigate effects of thermal stress we reduced egg survivorship to 0.63, but left all other parameters at control values. We also investigated a worst case scenario with male and female dehydration, no gel, and thermal stress. For all assays, we assumed that a single mating event would occur, rather than multiple, as the impact of multiple mating events has not been studied in this species. All values were derived from the previously described experiments. We determined the dominant eigenvalue of each matrix using the function “eigen” in R.

## Supplementary information

Supplementary Legends.

Supplementary Table S1.

Supplementary Table S2.

Supplementary Table S3.

Supplementary Table S4.

Supplementary Table S5.

Supplementary Table S6.

Supplementary Table S7.

Supplementary Table S8.

Supplementary Table S9.

Supplementary Table S10.

Supplementary Table S11.

Supplementary Table S12.

Supplementary Table S13.

Supplementary Table S14.

Supplementary Table S15.

Supplementary Figure S1.
