# Supplementary material for: Multi-level analysis of reproduction in an Antarctic midge identifies female and male accessory gland products that are altered by larval stress and impact progeny viability
Source: Sci Rep. 2020 Nov 13;10:19791. doi: 10.1038/s41598-020-76139-6 (PMC7666147; doi:10.1038/s41598-020-76139-6)
Supplement: Supplementary file 1 — Supplementary Legends. [file 41598_2020_76139_MOESM1_ESM.docx]

**Supplemental table 1.** RNA-seq results for complete *Belgica* *antarctica* gene set. Expression values are in transcripts per million. RNA-seq datasets are available under the following NCBI Bioprojects PRJNA174315 and PRJNA576639.

**Supplemental table 2**- BLAST results and expression results for female accessory gland specific *de novo* transcriptome against bacterial sequences from the NCBI nr database. RNA-seq datasets are available under the following NCBI Bioprojects PRJNA174315 and PRJNA576639.

**Supplemental table 3.** RNA-seq results for female-enriched *Belgica* *antarctica* gene set. Expression values are in transcripts per million. RNA-seq datasets are available under the following NCBI Bioprojects PRJNA174315 and PRJNA576639.

**Supplemental table 4.** RNA-seq results for male-enriched *Belgica* *antarctica* gene set. Expression values are in transcripts per million. RNA-seq datasets are available under the following NCBI Bioprojects PRJNA174315 and PRJNA576639.

**Supplemental table 5.** RNA-seq results for larvae-enriched *Belgica* *antarctica* gene set. Expression values are in transcripts per million. RNA-seq datasets are available under the following NCBI Bioprojects PRJNA174315 and PRJNA576639.

**Supplemental table 6.** RNA-seq results for female accessory gland-enriched *Belgica* *antarctica* gene set. Expression values are in transcripts per million. RNA-seq datasets are available under the following NCBI Bioprojects PRJNA174315 and PRJNA576639.

**Supplemental table 7.** RNA-seq results for male accessory gland-enriched *Belgica* *antarctica* gene set. Expression values are in transcripts per million. RNA-seq datasets are available under the following NCBI Bioprojects PRJNA174315 and PRJNA576639.

**Supplemental table 8.** WGCNA module results for female-enriched *Belgica* *antarctica* gene set. Expression values are in transcripts per million. RNA-seq datasets are available under the following NCBI Bioprojects PRJNA174315 and PRJNA576639.

**Supplemental table 9.** WGCNA module results for male-enriched *Belgica* *antarctica* gene set. Expression values are in transcripts per million. RNA-seq datasets are available under the following NCBI Bioprojects PRJNA174315 and PRJNA576639.

**Supplemental table 10.** WGCNA module results for larvae-enriched *Belgica* *antarctica* gene set. Expression values are in transcripts per million. RNA-seq datasets are available under the following NCBI Bioprojects PRJNA174315 and PRJNA576639.

**Supplemental table 11.** WGCNA module results for female accessory gland-enriched *Belgica* *antarctica* gene set. Expression values are in transcripts per million. RNA-seq datasets are available under the following NCBI Bioprojects PRJNA174315 and PRJNA576639.

**Supplemental table 12.** WGCNA module results for male accessory gland-enriched *Belgica* *antarctica* gene set. Expression values are in transcripts per million. RNA-seq datasets are available under the following NCBI Bioprojects PRJNA174315 and PRJNA576639.

**Supplemental table 13.** Transcript levels for gel-derived proteins from the *Belgica* *antarctica* gene set. Expression values are in transcripts per million. RNA-seq datasets are available under the following NCBI Bioprojects PRJNA174315 and PRJNA576639. Relative abundance of protein amounts was based on the number of protein fragments that matched specific genes.

**Supplemental table 14.** Transcript levels for transcription factors from the *Belgica* *antarctica* gene set. Expression values are in transcript per million. RNA-seq datasets are available under the following NCBI Bioprojects PRJNA174315 and PRJNA576639.

**Supplemental table 15**- Quantitative PCR primers used for the validation of RNA-seq result.

**Supplemental figure 1.** Protein gel used for proteomics (1D 4-12% Bis-Tris Invitrogen NuPage gel using MOPS buffer). Details included on the image.
