# Supplementary material for: Multi-level analysis of reproduction in an Antarctic midge identifies female and male accessory gland products that are altered by larval stress and impact progeny viability
Source: Sci Rep. 2020 Nov 13;10:19791. doi: 10.1038/s41598-020-76139-6 (PMC7666147; doi:10.1038/s41598-020-76139-6)
Supplement: Supplementary file 17 — Supplementary Figure S1. [file 41598_2020_76139_MOESM17_ESM.docx]

**Multi-level analysis of reproduction in an Antarctic midge identifies female and male accessory gland products that are altered by larval stress and impact progeny viability**

Geoffrey Finch, Sonya Nandyal, Carlie Perrieta, Benjamin Davies, Andrew J. Rosendale, Christopher J. Holmes, Josiah D. Gantz, Drew Spacht, Samuel T. Bailey, Xiaoting Chen, Kennan Oyen, Elise M. Didion, Souvik Chakraborty, Richard E. Lee, Jr., David L. Denlinger, Stephen F. Matter, Geoffrey M. Attardo, Matthew T. Weirauch, and Joshua B. Benoit

**
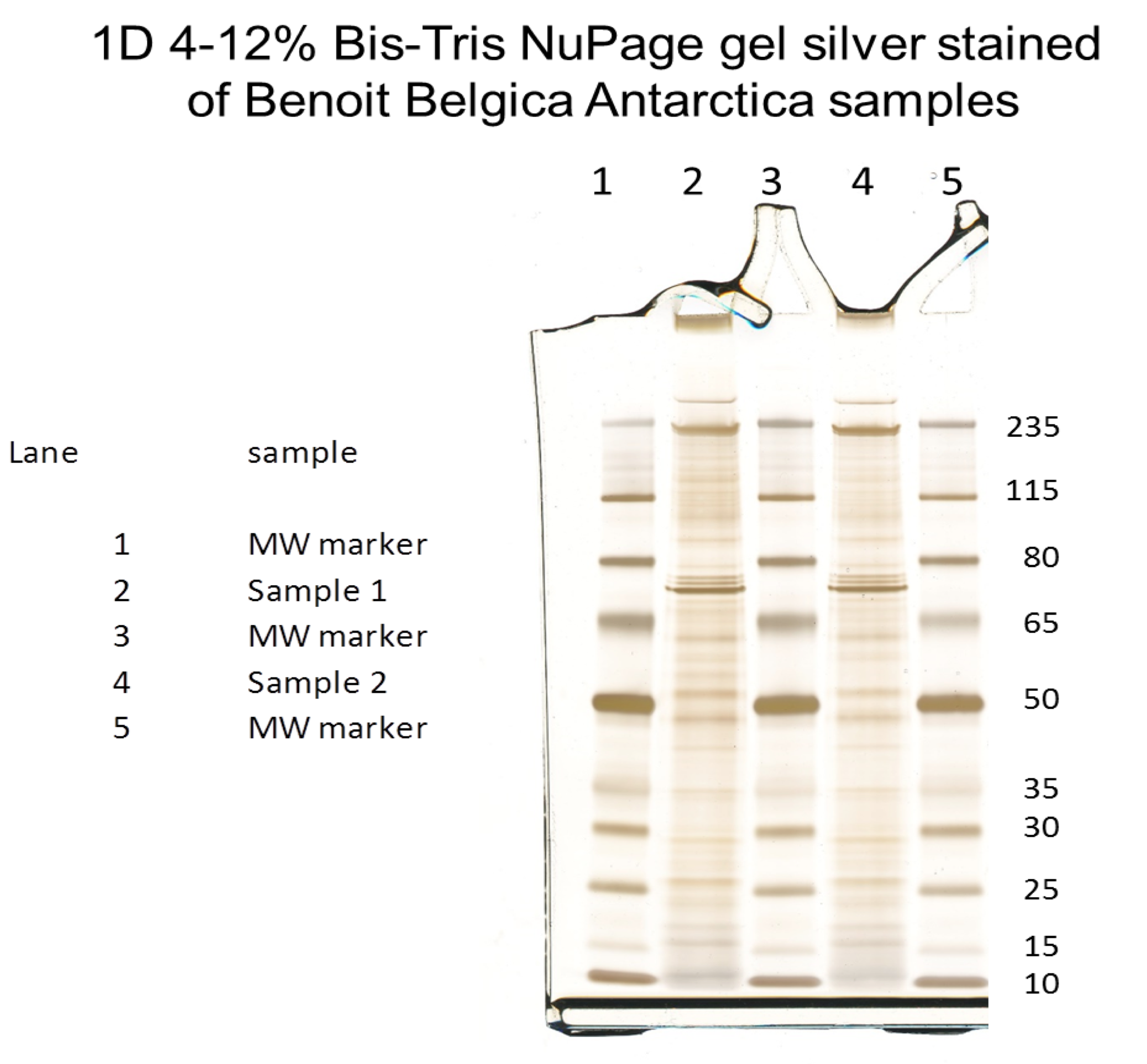
**

**Supplemental figure 1.** Protein gel used for proteomics (1D 4-12% Bis-Tris Invitrogen NuPage gel using MOPS buffer). Details included on the image.
